# A shift in lung macrophage composition is associated with COVID-19 severity and recovery

**DOI:** 10.1126/scitranslmed.abn5168

**Published:** 2022-09-14

**Authors:** Steven T. Chen, Matthew D. Park, Diane Marie Del Valle, Mark Buckup, Alexandra Tabachnikova, Ryan C. Thompson, Nicole W. Simons, Konstantinos Mouskas, Brian Lee, Daniel Geanon, Darwin D’Souza, Travis Dawson, Robert Marvin, Kai Nie, Zhen Zhao, Jessica LeBerichel, Christie Chang, Hajra Jamal, Guray Akturk, Udit Chaddha, Kusum Mathews, Samuel Acquah, Stacey-Ann Brown, Michelle Reiss, Timothy Harkin, Marc Feldmann, Charles A. Powell, Jaime L. Hook, Seunghee Kim-Schulze, Adeeb H. Rahman, Brian D. Brown, The Mount Sinai COVID-19 Biobank Team, Noam D. Beckmann, Sacha Gnjatic, Ephraim Kenigsberg, Alexander W. Charney, Miriam Merad

**Affiliations:** 1 The Precision Immunology Institute, Icahn School of Medicine at Mount Sinai, New York, NY 10029, USA.; 2 The Tisch Cancer Institute, Icahn School of Medicine at Mount Sinai, New York, NY 10029, USA.; 3 Department of Oncological Sciences, Icahn School of Medicine at Mount Sinai, New York, NY 10029, USA.; 4 Human Immune Monitoring Center, Icahn School of Medicine at Mount Sinai, New York, NY 10029, USA.; 5 Department of Genetics and Genomic Sciences, Icahn School of Medicine at Mount Sinai, New York, NY 10029, USA.; 6 Icahn Institute of Data Science and Genomics Technology, New York, NY 10029, USA.; 7 Department of Pathology, Molecular and Cell-Based Medicine, Icahn School of Medicine at Mount Sinai, New York, NY 10029, USA.; 8 Division of Pulmonary, Critical Care and Sleep Medicine, Department of Medicine, Icahn School of Medicine at Mount Sinai, New York, NY 10029, USA.; 9 Kennedy Institute of Rheumatology, Nuffield Department of Orthopaedics, Rheumatology and Musculo-skeletal Sciences, Botnar Research Centre, University of Oxford, Oxford OX3 7LD, UK.; 10 Global Health and Emerging Pathogens Institute, Department of Microbiology, Icahn School of Medicine at Mount Sinai, New York, NY 10029, USA.; 11 Icahn School of Medicine at Mount Sinai, New York, NY 10029, USA.; 12 Department of Psychiatry, Icahn School of Medicine at Mount Sinai, New York, NY 10029, USA.

## Abstract

Although it has been more than 2 years since the start of the coronavirus disease 2019 (COVID-19) pandemic, COVID-19 continues to be a worldwide health crisis. Despite the development of preventive vaccines, therapies to treat COVID-19 and other inflammatory diseases remain a major unmet need in medicine. Our study sought to identify drivers of disease severity and mortality to develop tailored immunotherapy strategies to halt disease progression. We assembled the Mount Sinai COVID-19 Biobank, which was composed of almost 600 hospitalized patients followed longitudinally through the peak of the pandemic in 2020. Moderate disease and survival were associated with a stronger antigen presentation and effector T cell signature. In contrast, severe disease and death were associated with an altered antigen presentation signature, increased numbers of inflammatory immature myeloid cells, and extrafollicular activated B cells that have been previously associated with autoantibody formation. In severely ill patients with COVID-19, lung tissue–resident alveolar macrophages not only were drastically depleted but also had an altered antigen presentation signature, which coincided with an influx of inflammatory monocytes and monocyte-derived macrophages. In addition, we found that the size of the alveolar macrophage pool correlated with patient outcome and that alveolar macrophage numbers and functionality were restored to homeostasis in patients who recovered from COVID-19. These data suggest that local and systemic myeloid cell dysregulation are drivers of COVID-19 severity and modulation of alveolar macrophage numbers and activity in the lung may be a viable therapeutic strategy for the treatment of critical inflammatory lung diseases.

## Modulation of macrophages

Understanding why some individuals develop severe disease after SARS-CoV-2 infection remains a high priority. Here, Chen *et al*. evaluated factors associated with disease severity and survival in samples from 600 individuals hospitalized with COVID-19 during 2020. The authors found that severe disease and death were associated with altered antigen presentation signatures, as well as a distinct macrophage profile in the peripheral blood. They also studied lung macrophages, finding that those with severe COVID-19 had increased inflammatory monocytes and monocyte-derived macrophage infiltration, with a corresponding decrease in the alveolar macrophage population. Together, these data suggest that restoring macrophage homeostasis may be a strategy for treating COVID-19.

## INTRODUCTION

Although there has been unprecedented success with the concurrent development of multiple highly effective vaccines against severe acute respiratory syndrome coronavirus 2 (SARS-CoV-2), there remains a critical need to develop additional immune therapies for vulnerable populations and severely ill patients. This is particularly true as multiple breakthrough variants continue to arise. In addition to coronavirus disease 2019 (COVID-19), there is a crucial need to characterize, modulate, and treat pathogenic inflammation associated with inflammatory illnesses, especially in older adult populations.

We assembled the Mount Sinai COVID-19 Biobank, which collected longitudinal blood samples from patients with COVID-19 (COVID^+^) and COVID-19–negative (COVID^−^) controls, and investigated local immune dynamics in the lungs of infected patients. A total of 583 COVID^+^ patients who were hospitalized at the Mount Sinai Hospital and 45 COVID^−^ volunteers from the Mount Sinai community were enrolled into the Mount Sinai COVID-19 Biobank. Serum and peripheral blood mononuclear cells (PBMCs) were collected from patients on time point 1 (T1), on average 14.8 ± 10.6 days post symptom onset (PSO). Samples were assigned time points according to approximately how many days after hospitalization the sample was collected (for example, 4 days after hospitalization = T4). Severely ill patients, who were hospitalized for greater than 2 weeks, had an additional sample collected 7 days later (T13). Severity scoring for each patient sample was assigned using clinical criteria designated by Mount Sinai Hospital, which overlapped with the World Health Organization clinical progression scale (1).

## RESULTS

### Proteomic characterization of COVID-19 serum samples reveals distinct immune patterns

**Fig. 1.**
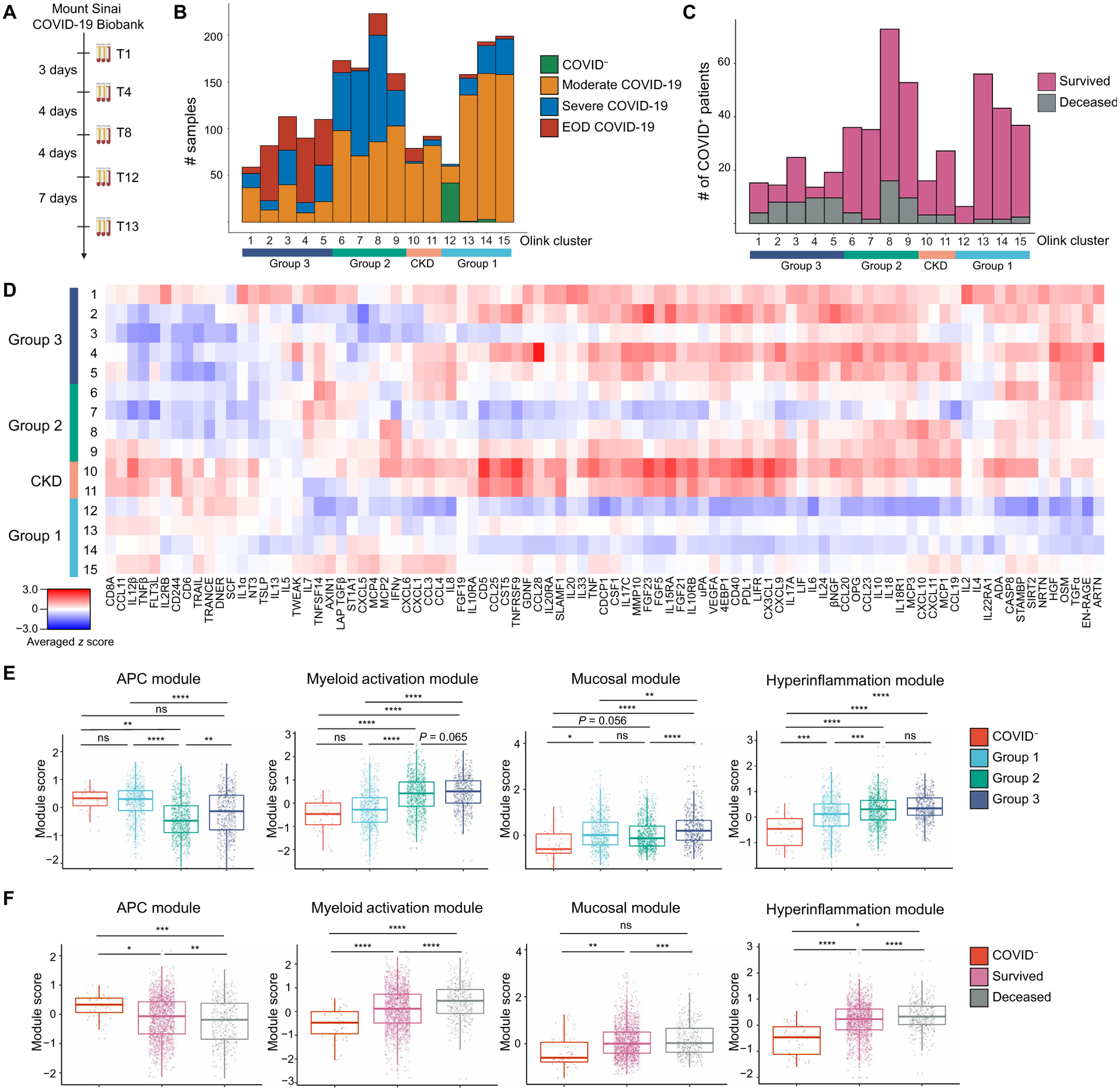
Proteomic characterization of COVID-19 serum reveals distinct immune patterns associated with disease severity and clinical outcome. (**A**) Mount Sinai COVID-19 Biobank serum collection scheme. (**B**) Histogram of patient samples across Olink clusters and Olink group is shown, denoted by clinical severity classification (*n* = 2001). (**C**) Histogram of first available COVID^+^ patient samples across Olink clusters and Olink group is shown, denoted by final clinical outcome (*n* = 583). (**D**) An averaged *z* score heatmap is shown of Olink inflammation panel analytes across Olink clusters. Olink clusters were grouped on the basis of clinical severity, projected outcome, and comorbidity distribution (*n* = 2001). (**E**) The boxplots showing Olink module score comparisons of all Olink samples by Olink group (*n* = 2001). (**F**) The boxplots show Olink module score comparisons of all Olink samples by the final clinical outcome (*n* = 2001). For box plots, each dot represents a patient sample; the center line indicates the median; box limits indicate the 25th and 75th percentile; whiskers indicate 1.5× inter-quartile range. The scheme in (A) was created with BioRender.com. COVID^−^ samples were obtained from healthy volunteers (B and D to F). Statistical significance in (E) and (F) is determined by two-way ANOVA with Tukey’s multiple comparisons correction. ns, not significant; *adj. *P* < 0.05; **adj. *P* < 0.01; ***adj. *P* < 0.001; ****adj. *P* < 0.0001.

To characterize the diversity of immune patterns in COVID^+^ patients, we measured 92 different cytokines on 1956 COVID^+^ and 45 COVID^−^ serum samples using the Olink inflammation panel. Instead of solely relying on clinical severity to group patients, we performed unsupervised clustering to unbiasedly sort serum samples into 15 different cytokine clusters (Fig. 1, A to D, and figs. S1 and S2). The majority of patients had one to four time points, which were distributed across all cytokine clusters (fig. S3, A to D). We found that the immune patterns were associated with clinical severity and final patient outcome, leading us to group them (data files S1 and S2 and table S1). Group 1 consisted of clusters 12 to 15 and was enriched in samples from COVID^−^ controls and patients with moderate COVID-19; group 2, which included clusters 6 to 9, was our largest and most heterogeneous group but was enriched in severe COVID-19 samples. In particular, clusters 8 and 9 had increased concentrations of interferon-γ (IFN-γ)–responsive and T helper type 1 (T_H_1) activation cytokines [such as IFN-γ; C-X-C motif chemokine ligands 9, 10, and 11 (CXCL9, CXCL10, and CXCL11); and interleukin-2 (IL-2)] compared to clusters 6 and 7 (2). Group 3, clusters 1 to 5, was enriched in severe COVID-19 with end organ damage (EOD) samples, as well as samples from patients who died from COVID-19.

Our unbiased clustering was not driven by sex, body mass index, or smoking status, but age and days PSO at time of sampling were higher in group 3 (fig. S3, E to I). Group 2 and group 3 patients had higher concentrations of C-reactive protein and D-dimer, indicating increased inflammation and hypercoagulability (fig. S3, J and K). Hypertension (HTN) and diabetes mellitus (DM) were common comorbidities within our cohort, especially in group 3 (fig. S3L). Clusters 10 and 11 were highly enriched in patients with chronic kidney disease (CKD), HTN, DM, and heart failure, leading us to group them into a distinct CKD group. Almost all patients received anticoagulation, and patients in groups 2 and 3 were more likely to receive steroids (fig. S3M). With the exception of C-C motif chemokine ligand 23 (CCL23) at the last collected time points of group 3 patients on enoxaparin, we did not observe any differences in cytokine concentrations between group 2 and group 3 patients who received steroids, norepinephrine, heparin, or enoxaparin at T1 or later time points (data file S3). We also found that many patient samples with similar clinical parameters were assigned to different clusters, indicating that clinical severity classification was unable to fully capture the diversity of immune patterns in COVID-19.

On the basis of the covariance patterns of the Olink cytokines (fig. S4), we identified four protein modules and calculated module scores for each group. The antigen-presenting cell (APC) module, which included proteins associated with antigen presentation, dendritic cells (DCs), and T cell activation [tumor necrosis factor (TNF)–related apoptosis-inducing ligand, TNF-related activation-induced cytokine, IL-12β, FMS-like tyrosine kinase 3 ligand (Flt3L), and TNF-β], scored higher in COVID^−^ controls and patients with moderate disease (3–5). Next, we identified a core group of four cytokines released by activated monocytes and neutrophils, [transforming growth factor–α (TGF-α), hepatocyte growth factor (HGF), oncostatin M (OSM), and S100 calcium binding protein A12 (S100A12/EN-RAGE)], which were enriched in patients with severe or EOD COVID-19 and grouped them into a myeloid activation module. Signaling by these cytokines has been associated with proinflammatory cytokine secretion, fibroblast activation, and fibrosis (6–11). The mucosal module, which included T_H_17 and barrier defense cytokines (IL-17A, IL-17C, CCL20, CCL28, and IL-33), and the hyperinflammation module, which included inflammatory cytokines (TNF, IL-6, IL-8, IL-10, IL-18, CXCL10, and monocyte chemo-attractant protein–3), were enriched in patients with severe or EOD disease (12, 13). We grouped these analytes into a mucosal module and a hyperinflammation module, respectively.

APC module scores were higher in COVID^−^ controls and group 1 but were reduced in groups 2 and 3. In contrast, myeloid activation, mucosal, and hyperinflammation module scores were higher in groups 2 and 3 (Fig. 1E). Comparison of module scores by outcome showed that patients who survived had higher APC module scores, whereas patients who died had higher myeloid activation, mucosal, and hyperinflammation module scores (Fig. 1F). Many of these observations held even when we compared only the first time points for each patient, suggesting that module scores could be used early on during a patient’s hospitalization to predict clinical outcome (fig. S5, A and B). Univariate logistic regression analysis showed that Olink grouping, APC module, and myeloid activation module scores were good predictors of survival (fig. S5C).

To determine the stability of these cytokine patterns, we performed discrete time Markov chain analysis on all COVID^+^ serum samples to determine the probability of transition between clusters across successive samples, irrespective of past or future states (fig. S5D) (14). Between time points, group 1 patients had the highest probability of transitioning to other group 1 clusters and the highest probability of survival. Group 2 patients also had a high probability of transitioning to group 1, clusters 13 to 15, but had an increased probability of death compared to group 1. Patients with CKD had about 50% probability of survival or death between time points. Group 3 patients rarely transitioned to other clusters and had the highest probability of dying.

Our proteomic analyses highlight the heterogeneity of immune states in COVID-19 that remained stable over time, despite medical intervention, and the value of using our Olink grouping and module scores to predict clinical outcome. The averaged cytokine values by cluster were highly correlated not only within groups (fig. S6A) but also across time points, further supporting the stability of patient immune states (fig. S6, B and C). The heterogeneity revealed by our clustering underscores the limitations of solely using clinical severity parameters to stratify patients for treatment. For example, whereas CKD and group 3 patients would likely benefit from broad immune suppression and targeted therapies like IL-6 blockade, these same treatments are unlikely to show the same effect in group 2 patients and may instead hinder protective antiviral adaptive immune responses. On the other hand, all patients would likely benefit from therapies to boost their APC response, such as administration of Flt3L, to increase the number of DCs for T cell priming and activation.

### Myeloid cell dysregulation underlies COVID-19 severity

**Fig. 2.**
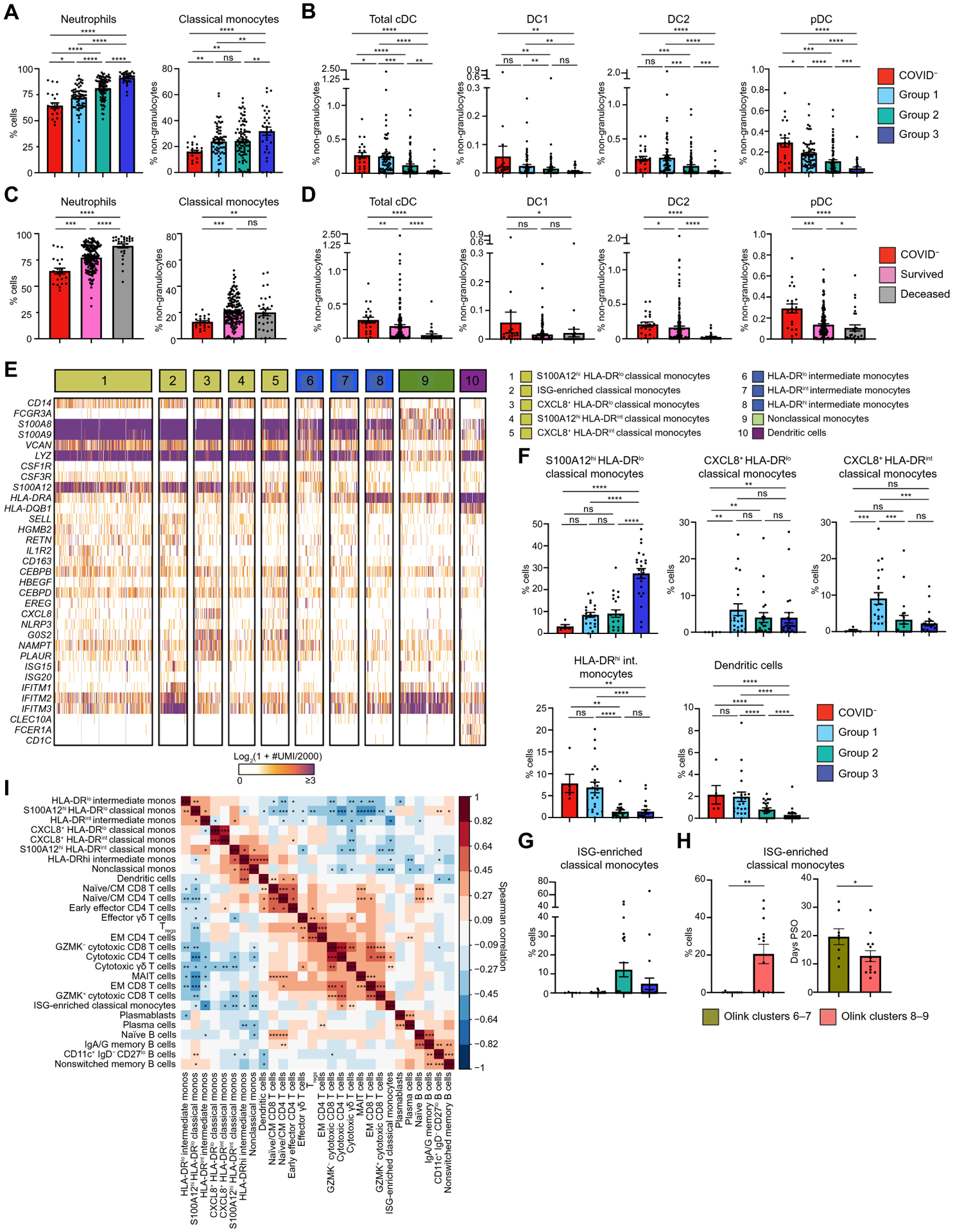
Immature inflammatory myeloid cells are associated with increased COVID-19 severity. (**A**) The frequency of neutrophils (% cells) and classical monocytes (% non-granulocytes) in whole blood were measured by CyTOF and separated by Olink group (*n* = 206). (**B**) DC population frequencies (% non-granulocytes) in whole blood were measured by CyTOF and separated by Olink group (*n* = 206). Conventional DC (cDC), conventional type 1 DC (DC1), conventional type 2 DC (DC2), and plasmacytoid DC (pDC) are shown. (**C**) Neutrophils (% cells) and classical monocyte frequencies (% non-granulocytes) in whole blood are shown on the basis of the final clinical outcome and were measured by CyTOF (*n* = 214). (**D**) DC population frequencies (% non-granulocytes) in whole blood are shown on the basis of the final clinical outcome and were measured by CyTOF (*n* = 214). (**E**) The heatmap shows unique molecular identifier (UMI) counts of selected genes from myeloid cell scRNAseq clusters from PBMCs. (**F**) scRNAseq cluster cell frequencies in indicated Olink groups are shown as percent of cells by Olink group (**G**) Frequencies of ISG-enriched classical monocytes are shown clustered by Olink group (*n* = 75). (**H**) ISG-enriched classical monocyte cell frequencies and days PSO are shown separated by clusters 6 and 7 versus clusters 8 and 9 (*n* = 10). (**I**) The matrix heatmap shows Spearman correlation coefficients between identified scRNAseq PBMC cell clusters (*n* = 81). Monos, monocytes. (*P < 0.05; **P < 0.01; ***P < 0.001). For bar graphs (A to D and F to H), each dot represents a patient sample. COVID^−^ samples were obtained from healthy volunteers (A to G and I). Statistical significance (A to D and F and G) was determined by Kruskal-Wallis followed by the multiple comparisons test with false discovery rate correction. ns, not significant; *q < 0.05; **q < 0.01; ***q < 0.001; ****q < 0.0001. Statistical significance in (H) was determined by the Mann-Whitney test; *P < 0.05; **P < 0.01.

We performed cytometry by time of flight (CyTOF) on whole blood samples from hospitalized COVID^+^ patients and COVID^−^ volunteers to measure circulating immune cell composition and its association with Olink groups (fig. S7 and data file S4). Consistent with prior studies, we found that neutrophils and classical monocytes were increased, whereas all DC populations were decreased in more severe disease (Fig. 2, A and B). Grouping patient samples by outcome showed that patients who died from COVID-19 had increased numbers of neutrophils and decreased total conventional DC (cDC), conventional type 2 DC (DC2), and plasmacytoid DC (pDC) (Fig. 2, C and D). CyTOF also showed lymphopenia of CD4 and CD8 T cell populations in group 2 and group 3 patients (fig. S8, A and B) (15, 16).

To dissect the heterogeneity of circulating immune cells in an unbiased manner, we performed single-cell RNA sequencing (scRNAseq) on 81 PBMC samples from 39 COVID^+^ patients and 6 COVID^−^ volunteers (table S2 and data file S5). After downsampling, integration, batch correction, and removal of doublet cells, unsupervised clustering revealed discrete subsets of circulating immune cells (Fig. 2E). We identified a cluster of classical monocytes that highly expressed RAGE ligands *S100A12* (EN-RAGE), *S100A8*, and *S100A9* but lowly expressed human leukocyte antigen (HLA) molecules. Low expression of HLA and *CSF1R* with concurrent high expression of granulocyte/monocyte precursor genes (such as *CSF3R*, *CEBPB*, and *CEBPD*) indicated that this cluster likely represents immature cells arising from granulocyte-monocyte progenitors (17–19). This cluster, which we named S100A12^hi^ HLA-DR^lo^ classical monocytes, was found at higher relative frequencies in group 3 (Fig. 2F).

We identified three clusters of inflammatory immature monocytes that highly expressed *S100A12*, inflammasome protein *NLRP3*, and oxidative stress marker *NAMPT* (20, 21). We distinguished these three clusters by relative *CXCL8* and *HLA-DR* expression, leading us to name them CXCL8^+^ HLA-DR^lo^ classical monocytes, S100A12^hi^ HLA-DR^int^ classical monocytes, and CXCL8^+^ HLA-DR^int^ classical monocytes. Next, we identified three clusters of intermediate monocytes with varying degrees of HLA-DR expression. Among these subsets, HLA-DR^hi^ intermediate monocytes were found at higher relative frequencies in group 1 patients relative to groups 2 and 3. In accordance with our CyTOF results, DCs were decreased in more severely ill Olink groups. Together, our data support previous work that immature inflammatory myeloid cells, likely arising from emergency myelopoiesis, are associated with increased COVID-19 severity (18, 19).

We also identified a cluster of classical monocytes expressing type I IFN-stimulated genes (ISG) (including *ISG15, ISG20*, and *IFITM1* to *IFITM3*) primarily in group 2 patients (Fig. 2G). Given the differences that we saw in T cell activation cytokines by Olink, we stratified this cluster of ISG-enriched classical monocytes by clusters 6 and 7 versus clusters 8 and 9. This group of monocytes was only found in patients in clusters 8 and 9, which could be due to transient or delayed IFN signaling captured by the earlier sampling of these patients (Fig. 2H) (22, 23).

Next, we performed unbiased clustering to identify lymphocyte clusters. Naïve/central memory (CM) CD4 and CD8 T cells expressed *CCR7*, *IL7R*, *LDHB*, *LTB*, *LEF1*, and *TCF7* (fig. S8, C to E) (24, 25). Naïve/CM CD4 T cells were found at higher frequencies in COVID^−^ and group 1 patients. We identified early effector CD4 T cells by low expression of *KLRB1*, *CCL5*, and *GZMM*; a cluster of T regulatory cells (T_regs_); and mucosal-associated invariant T (MAIT) cells by *KLRB1*, *NKG7*, *GZMK*, *GZMA*, and *CCL5* expression. Effector memory (EM) CD4 and CD8 T cells had intermediate expression of *IL7R* and *LTB* and low expression of *LEF1*, *CCR7*, and *TCF7*. In addition, we identified Granzyme K (GZMK^+^), GZMK^−^ cytotoxic CD8 T cells, and cytotoxic CD4 T cells as well as cytotoxic and effector γδ T cells based on granzyme and *GNLY* expression. Among B cells, we identified plasmablasts and plasma cells by *CD38*, *CD27*, and *MZB1* expression; plasma cells were further distinguished by *PRDM1* expression (fig. S8F) (26). Naïve B cells were identified by high expression of *IGHD*^+^ and *IGHM*^+^. We also noted higher frequencies of CD11c^+^ immunoglobulin D^−^ (IgD^−^) CD27^lo^ B cells, which are thought to be extrafollicular or polyreactive B cells that produce pathogenic autoantibodies in group 3 patients relative to group 1 and group 2 patients (fig. S8G) (27–29).

### Integration of circulating immune cell phenotypes and serum proteomics

To determine how these different immune cell populations might be interacting, we performed Spearman correlation analysis on the scRNAseq cell frequencies. We found that the frequencies of S100A12^hi^ HLA-DR^lo^ classical monocytes and other HLA-DR^lo^ immature monocyte clusters were negatively correlated with the ISG-enriched classical monocytes, DCs, and cytotoxic T cells but were positively correlated with CD11c^+^ IgD^−^ CD27^lo^ B cells (Fig. 2I). In contrast, DCs were positively correlated with naïve/CM CD4, CD8, and early effector CD4 T cells.

**Fig. 3.**
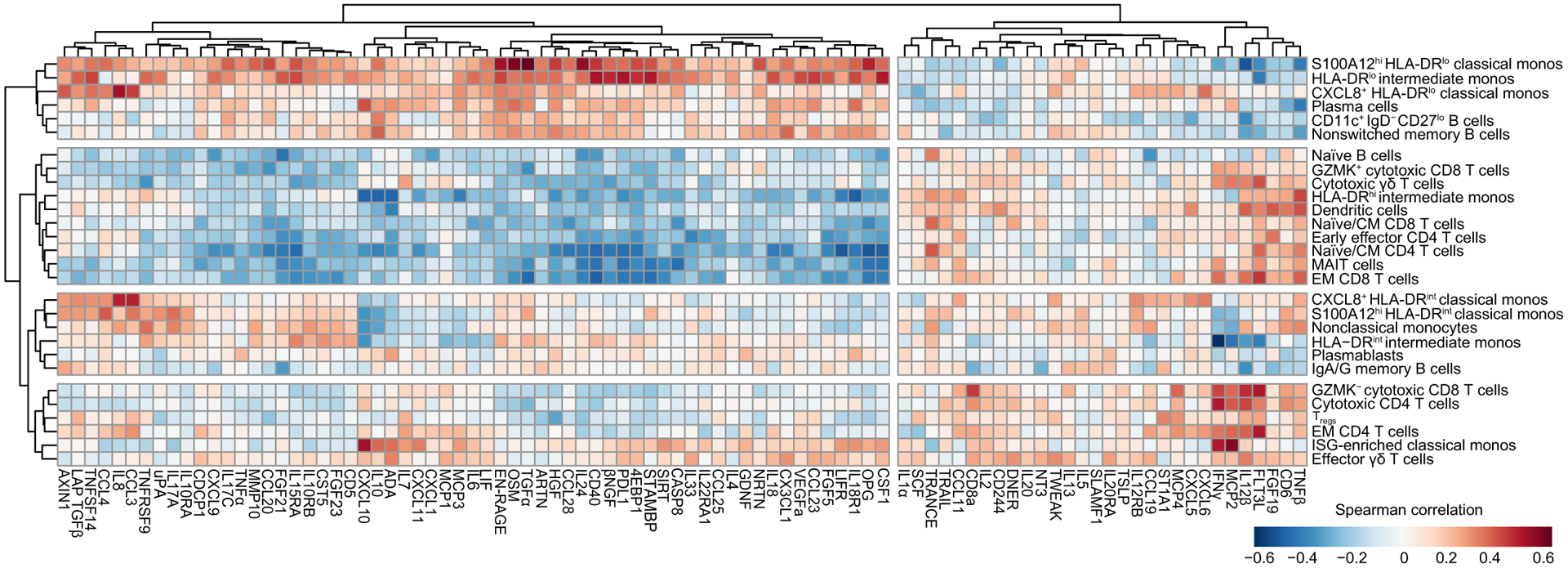
Integrated analysis of scRNAseq cluster frequencies and Olink analyte abundance in serum reveals distinct immune responses to COVID-19. Shown is a matrix heatmap of Spearman correlation coefficients between identified scRNAseq PBMC cell clusters (*y* axis) and Olink analyte–normalized concentrations (*x* axis) in serum. Axes are ordered by hierarchical clustering. Monocytes, monos.

Following this, we correlated scRNAseq cell composition and Olink proteomics and used a hierarchical clustering to explore the relationship between cell subsets and serum cytokines (Fig. 3). Immature HLA-DR^lo^ myeloid cells, CD11c^+^ IgD^−^ CD27^lo^ B cells, and nonswitched memory B cell frequencies positively correlated with myeloid activation, mucosal, and hyperinflammatory module cytokine concentrations and strongly negatively correlated with APC module cytokines. This pattern corresponded most closely with group 3 patients, who had increased circulating immature myeloid cells and higher serum concentrations of these inflammatory cytokines. In contrast, DCs clustered together with naïve/CM CD4 and CD8 T cells, GZMK^+^ cytotoxic CD8 T cells, cytotoxic γδ T cells, MAIT cells, and naïve B cells. These cell types were positively correlated with APC module cytokines and negatively correlated with myeloid activation, mucosal, and hyperinflammation module cytokines. This pattern most closely corresponded to group 1 patients, who had higher serum concentrations of APC–T cell activating cytokines and greater numbers of circulating effector T cells.

We also identified two other patterns of cell-cytokine profiles. HLA-DR^int^ monocyte populations clustered together with nonclassical monocytes, plasmablasts, and IgA/IgG memory B cells. These cell types were negatively correlated with IFN-γ, IL-12β, CXCL10, and CXCL11. In contrast, ISG-enriched classical monocytes clustered with EM CD4 T cells, GZMK^−^ cytotoxic CD8 T cells, and effector γδ T cells. These cell populations were positively correlated with IFN-γ, IL-12β, CD8α, IL-2, and Flt3L and negatively correlated with myeloid activation and mucosal module cytokines.

This integrated analysis revealed four distinct types of immune response to COVID-19. First, group 1 patients, who had higher numbers of more mature HLA-DR^hi^ myeloid cells, had correspondingly higher numbers of effector and cytotoxic T cell populations, higher serum concentrations of APC and T cell–activating cytokines, and decreased serum concentrations of inflammatory, tissue-damaging cytokines. These patients tended to have a milder course of COVID-19 and were more likely to survive. On the other end of the spectrum, group 3 patients, who had high numbers of immature HLA-DR^lo^ myeloid cells with limited antigen presentation capability, were likely unable to mount strong T cell responses and were instead likely more reliant on humoral control of infection. Immature myeloid cells in these patients may have predominated due to increased inflammatory cytokines that drove emergency myelopoiesis (30, 31). These immature myeloid cells may have further contributed to hyperinflammation by producing tissue-damaging cytokines and reactive oxygen species, leading to a cycle of lymphopenia, suppressed or delayed adaptive immunity, poorer control of virus infection, and increased inflammation. This inflammation may have been further exacerbated by extrafollicular CD11c^+^ IgD^−^ CD27^lo^ B cells that produced autoantibodies, leading to autoimmune-mediated inflammation (32, 33). Consequently, these patients had the lowest rates of survival. Patients with an earlier type I IFN response, as well as those who had higher numbers of mature HLA-DR^int/hi^ monocytes and DCs, may have been better protected against disease progression and morbidity, because they were able to mount an earlier, more productive adaptive T cell response.

### Loss of alveolar macrophages and phenotypic changes in COVID-19 lung microenvironment

**Fig. 4.**
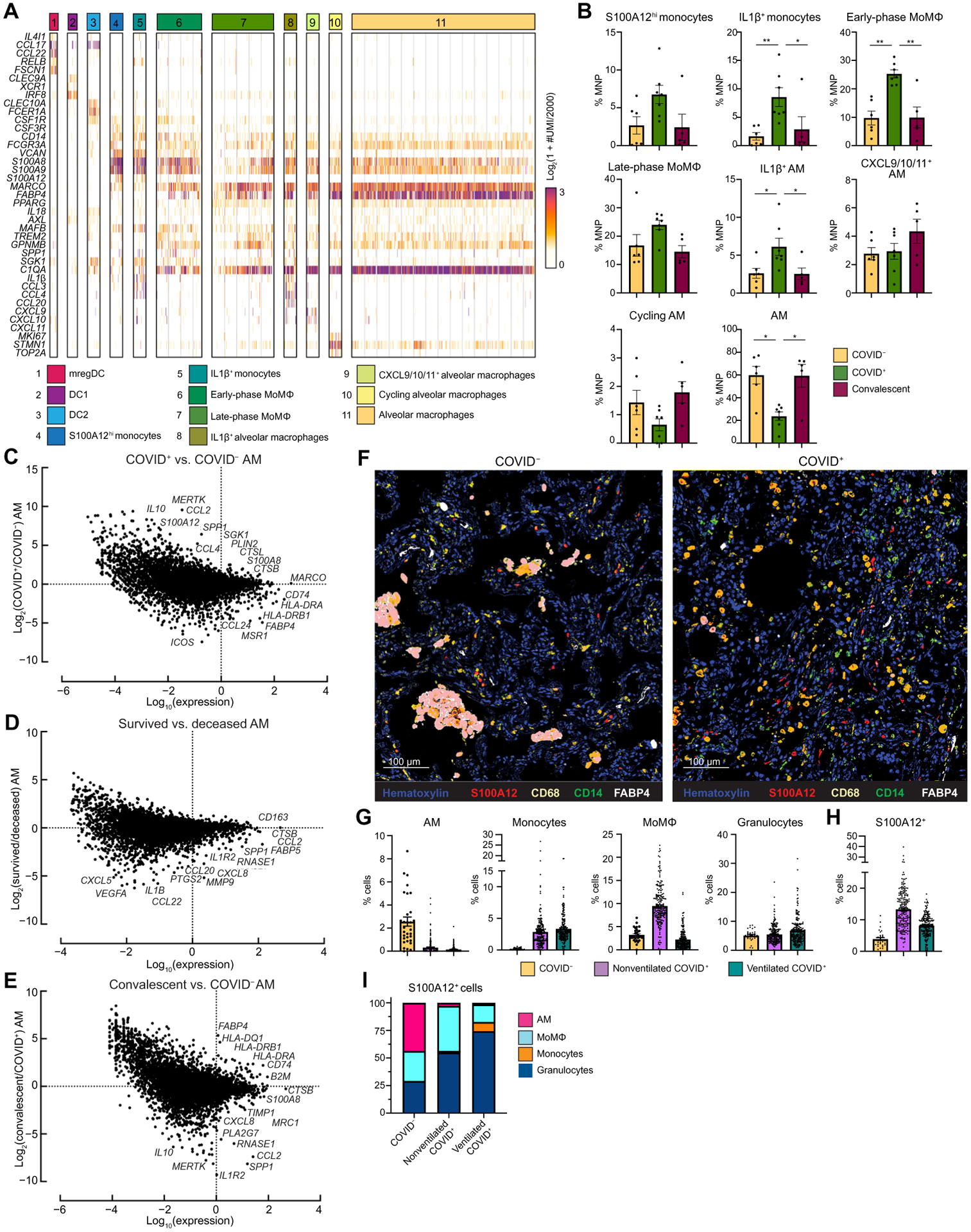
AM loss and phenotypic changes are associated with COVID-19. (**A**) The heatmap shows UMI counts of selected genes from myeloid cell scRNAseq clusters from BAL. (**B**) scRNAseq cluster cell frequencies are shown as percent of mononuclear phagocytes (MNP) in COVID^−^, COVID^+^, or convalescent patient BAL (*n* = 18). COVID^−^ and convalescent samples obtained from Mount Sinai Hospital patients (C to E). Differential gene expression is shown between alveolar macrophages (AMs) from COVID^+^ and COVID^−^ patients (**C**), AMs from patients that survived versus deceased (**D**), and AMs from convalescent and COVID^+^ patients (**E**). (**F**) Overlaid, pseudo-colored MICSSS images of COVID^+^ and COVID^−^ lungs are shown. Samples were stained for S100A12, CD68, CD14, FABP4, and hematoxylin (*n* = 5). (**G**) Quantification of myeloid cells in MICSSS images is shown as percent of cells. AMs were defined as FABP4^+^CD68^+^ cells; monocytes were defined as CD14^+^ cells; MoMΦ were defined as CD14^+^CD68^+^ cells; and granulocyte cells were defined as CD66b^+^ cells or by hematoxylin staining and morphology. (**H**) Quantification of S100A12^+^ cells in MICSSS images is shown as percent of cells in the COVID^−^ patient (*n* = 1), nonventilated COVID^+^ patients (*n* = 2), or ventilated COVID^+^ patients (*n* = 2). (**I**) Distribution of S100A12^+^ cells by cell type in COVID^−^, nonventilated, or ventilated COVID^+^ lungs is shown. For bar graphs, each dot represents a patient sample (B) or quantification of single MICSSS region of interest (G and H). Statistical significance in (B) was determined by the Kruskal-Wallis test followed by the multiple comparisons test with false discovery rate correction. *q < 0.05; **q < 0.01.

To characterize immune cell dynamics in the local lung microenvironment, we acquired bronchoalveolar lavage (BAL) samples from intubated patients with EOD, COVID^−^ controls, and convalescent patients who recovered from COVID-19 and performed scRNAseq (table S3 and data file S6). COVID^−^ and convalescent BAL samples were acquired from nonhospitalized patients undergoing bronchos-copy as part of cancer or lung disease screening. Similar to what we found in circulation, we identified a cluster of S100A12^hi^ monocytes that also highly expressed *S100A8* and *S100A9* (Fig. 4, A and B). COVID^+^ BAL had higher relative frequencies of both inflammatory IL-1β^+^ monocytes and IL-1β^+^ alveolar macrophages (AMs) that highly expressed *IL1B*, *CCL3*, and *CCL4*. Early-phase monocyte-derived macrophages (MoMΦ) highly expressed MoMΦ-associated genes *SGK1*, *MAFB*, *TREM2*, and *GPNMB* relative to late-phase MoMΦ and were increased in COVID^+^ patients compared to COVID^−^ and convalescent patients. Late-phase MoMΦ had higher expression of AM-associated genes (such as *MARCO* and *FABP4*) compared to early-phase MoMΦ, indicating further differentiation toward a resident tissue macrophage (RTM) phenotype. We found that the relative frequency of AMs, the RTM of the lung, was decreased in COVID^+^ patients compared to COVID^−^ patients but restored to homeostatic numbers in convalescent patients. When stratified by age, single-cell analysis of normal lung tissue from a cohort of patients with untreated early stage non–small cell lung cancer showed decreased AM in older (greater than 70 years old) patients and increased inflammatory MoMΦ, therefore indicating baseline differences in lung MNP composition in older adults (fig. S9A) (34). At baseline in COVID^−^ and convalescent individuals, the relative frequency of AMs was not affected by sex or by presence of malignancy (fig. S9, B and C). We did not observe a difference in AM frequency by age among our BAL cohort, although this was likely due to our small sample size (fig. S9D).

In addition to the decrease of the AM pool, AMs from COVID^+^ patients had higher expression of inflammatory RAGE ligands [*S100A12* (EN-RAGE) and *S100A8*] and monocyte chemokines *CCL2* and *CCL4*, whereas AMs from COVID^−^ individuals had higher expression of antigen presentation genes (*HLA-DRA*, *HLA-DRB1*, and *CD74*) and canonical AM markers (*MARCO*, *MSR1*, and *FABP4*) (Fig. 4C). These data suggested that AMs from COVID^+^ patients contributed to local inflammation, recruited monocytes from circulation, and were less proficient at antigen presentation to T cells. AMs from deceased COVID^+^ patients also had higher expression of neutrophil chemokines (*CXCL*5 and *CXCL8*), monocyte chemokine *CCL2*, and inflammatory cytokines (*IL1B* and *CCL22*) compared to AMs from COVID^+^ patients who survived (Fig. 4D). Previous studies reported deficits in AM phagocytosis, antigen presentation, and wound healing ability with increased age (35, 36). Comparison of AMs from convalescent and COVID^+^ patients showed higher expression of class I and II antigen presentation genes after recovery from COVID-19, thereby suggesting that AM numbers and functionality were restored to baseline in recovered patients (Fig. 4E). We did not find any gene expression differences between COVID^−^ and convalescent AMs (fig. S9E).

Spearman correlation of BAL cell population frequencies showed that inflammatory myeloid cells, S100A12^hi^ monocytes, IL-1β^+^ monocytes, MoMΦ, and IL-1β^+^ AMs were positively correlated with each other but negatively correlated with AMs, cytotoxic T cells, and T_regs_ (fig. S9, F and G). COVID^+^ BAL also had decreased T_regs_, indicating that local hyperinflammation may be partly due to loss of immunosuppressive regulation (fig. S9H). Together, these data suggest that the loss of AMs, due to either excessive inflammation or direct SARS-CoV-2 infection, and a decrease in their capacity to present antigen to recruit and prime T cells may have contributed to uncontrolled viral replication and tissue damage (37). Inflammatory IL-1β^+^ AMs and IL-1β^+^ monocytes may have further exacerbated lung inflammation by recruiting inflammatory immature myeloid cells from the periphery. Furthermore, older patients may be predisposed to severe COVID-19 due to decreased AM numbers and functionality, as well as increased infiltration of inflammatory lung MoMΦ at baseline.

We confirmed these findings on autopsy lung samples from COVID^+^ patients obtained 10.1 ± 6.2 hours postmortem using multiplexed immunohistochemical consecutive staining on single slide (MICSSS) (table S4) (38). Here, we again saw a depletion of AMs and an influx of monocytes, MoMФ, and granulocytes in COVID^+^ lungs compared to a COVID^−^ organ donor lung autopsy control (Fig. 4, F and G, and fig. S9I). Comparison between COVID^−^ and COVID^+^ patients showed increased frequencies of S100A12 (EN-RAGE)^+^ cells (Fig. 4H). These changes were not simply due to ventilation, because similar results were found in ventilated and nonventilated patients. We also observed a shift in the expression and localization of S100A12 from AMs in the alveolar air spaces of COVID^−^ lungs to expression by infiltrating monocytes, MoMΦ, and granulocytes in COVID^+^ lung interstitium (Fig. 4I). In line with the BAL scRNAseq, COVID^+^ lungs from both nonventilated and ventilated patients had decreased T_regs_ compared to control (fig. S9J). These data suggest that the loss of AMs and lung T_regs_ in COVID-19 may have led to an inability to resolve inflammation and to initiate tissue repair even after virus clearance, leading to autonomous inflammation that contributed to morbidity.

## DISCUSSION

In this work, we presented systemic and lung high-dimensional immunophenotyping on one of the largest single-center COVID-19 cohorts to date, which was collected during the height of the COVID-19 pandemic in New York City. We found that patients with moderate disease had increased numbers of circulating DCs and effector and cytotoxic T cells, increased serum concentrations of cytokines associated with APC function, and reduced concentrations of cytokines associated with myeloid activation, mucosal damage, and hyperinflammation. In contrast, severely ill patients had reduced DCs and effector and cytotoxic T cells and lower serum concentrations of cytokines associated with APC function and were enriched in immature inflammatory monocytes producing *S100A12* (EN-RAGE). Severely ill patients also had higher serum concentrations of cytokines associated with myeloid activation, mucosal damage, and hyperinflammation.

We found that lung tissue–resident AMs were profoundly altered in numbers and functionality in severe COVID-19. AMs from COVID^+^ patients expressed higher concentrations of inflammatory cytokines and had decreased expression of HLA class I/II genes compared to AMs from COVID^−^ patients, indicating a decrease not only in AM numbers but also in their antigen presentation capability. AMs from deceased COVID^+^ patients were also more inflammatory and expressed higher concentrations of neutrophil and monocyte chemokines compared to AMs from surviving patients, thereby implicating an additional role for AMs in perpetuating tissue-damaging inflammation and recruitment of immature inflammatory myeloid cells from the periphery. Depletion and alteration of the AM pool may be a consequence of direct infection by SARS-CoV-2, leading to the activation of inflammatory pathways and pyroptosis (39). At the same time, alveolar type II (AT2) cells, the primary angiotensin-converting enzyme 2 expressing cells in the lung and primary target of SARS-CoV-2, also produce granulocyte-macrophage colony-stimulating factor (GM-CSF) and have a nonredundant role in maintaining AMs in the lung (40). Thus, AM depletion may be multifactorial due to not only inflammation or virus infection but also decreased GM-CSF in the alveolar milieu. In addition to their role as first responders to pathogens in the lung, AMs play a key role in lung homeostasis, resolution of inflammation, and tissue repair (41). This may explain why older patients, who at baseline have decreased AMs and increased inflammatory MoMΦ, are predisposed to increased disease severity.

During development and under homeostatic conditions, RAGE signaling in AT1 cells helps maintain alveolar architecture and lung compliance but is also a known activator of nuclear factor κB signaling (42). We hypothesize that loss of AM-derived RAGE ligand signaling from *S100A8/A9* and *S100A12* (EN-RAGE) in the AT1 cells that line the alveolar air space may lead to defects in physiologic gas exchange. Furthermore, the shift of RAGE ligand production from AMs in alveoli to infiltrating monocytes and MoMΦ in the lung interstitium may exacerbate lung injury and vascular leakage, thereby leading to increased immature myeloid cell recruitment and infiltration. Increased inflammation has been implicated in defective transdifferentiation of AT2 to AT1 cells, which may inhibit reepithelialization and maintenance of alveolar barrier integrity (43).

Our study had several limitations. First, our Olink and PBMC scRNAseq COVID^−^ individuals were younger than our COVID^+^ patients due to the difficulty of acquiring samples from older COVID^−^ individuals during the pandemic. This limited how broadly we could apply our conclusions to the population at large and led us to focus our analysis mostly on the differences between patient groups while using our COVID^−^ group as a technical control. Second, although we did not find many differences in cytokine concentrations from various treatments, we cannot exclude the possibility that these treatments or combinations of medications affected our Olink clustering. However, because our clustering matched closely with clinical severity, it is also to be expected that certain standard-of-care therapies such as norepinephrine or anticoagulation would be enriched in group 2 or group 3 patients. Our study was not designed or powered to test the effects of different medications on the immune response, and such analyses would be further confounded, because patients were receiving multiple treatments, many of which were experimental, early on in the COVID-19 pandemic. Our cytokine analyses were also limited to serum, which does not directly reflect the local tissue immune milieu these treatments may have affected. Moreover, we did not have access to completely treatment-naïve patients in each group, making it difficult to properly control for the effects of medications on circulating cytokine concentrations. Third, we presented immune cell composition as cell frequencies rather than raw cell counts due to the technical limitations of cell capture and pooled sample acquisition in CyTOF and scRNAseq. Therefore, the changes in cell composition should be most appropriately regarded as changes in relative frequency. We tried to address this limitation by using orthogonal methods of analyzing immune cell composition, but further studies will be required to validate changes in absolute cell number. Fourth, although we hypothesized that systemic and local changes in myeloid composition as well as decreased expression of antigen presentation genes likely resulted in defective or delayed adaptive immunity, our data are correlative and hypothesis generating, as we did not have access to antigen-specific T and B cell reagents to validate these conclusions. Fifth, our BAL cohort is much smaller than our PBMC cohort. We were only able to collect a few samples of BAL from COVID^+^ patients due to potential exposure of clinical staff during sample collection. In addition, COVID^−^ BAL samples were acquired as part of clinical screening for diagnosis of cancer or other lung diseases. Several samples from our COVID^−^ and convalescent patients were positive for malignancy, which may have affected their immune cell populations at baseline. Yet, we also believe that this makes our conclusions regarding AM depletion and influx of inflammatory monocytes in COVID-19 even more compelling, as we were able to detect these differences even when comparing to a group primarily consisting of patients with cancer.

Despite these limitations, our study provides insight into the systemic and local immune cytokine and cell dynamics that may contribute not only to COVID-19 severity and recovery but also to other infections and inflammatory lung diseases. Our data suggest that preserving and restoring AM numbers early during infection, such as through nebulized delivery of GM-CSF, may be a valid therapeutic strategy to protect airway integrity and to initiate an early innate and adaptive immune response (44). Future work should also focus on limiting the expansion and recruitment of immature inflammatory myeloid cells from the periphery, as well as depletion of inflammatory MoMΦ to control tissue damaging inflammation in the lung.

## MATERIALS AND METHODS

### Study design

The goal of our study was to identify drivers of COVID-19 severity and death to support the identification and development of tailored immunotherapy strategies to halt disease progression. We performed high-dimensional immunophenotyping on patients who were hospitalized with COVID-19 at the Mount Sinai Hospital from March to December 2020. Patients were not randomized or blinded to investigators as their COVID-19 status was relevant for analysis. Sample size for power analysis was not predetermined for our study. All proteomics, transcriptomics, and imaging data, including outliers, were used for analysis except for measurements of Olink analyte matrix metalloproteinase–1, which were excluded because of technical batch effects. In this study, we characterized serum inflammation patterns using the Olink platform, which allowed us to detect 92 different proteins in 583 COVID^+^ patients (1956 COVID^+^ and 45 COVID^−^ volunteer serum samples). To understand the diversity of immune patterns, we performed an unbiased clustering analysis and identified immune patterns that correlated with disease severity, comorbidities, and patient outcome. We grouped immune patterns based on these clinical parameters and calculated protein module scores based on the covariance patterns of different cytokines. Next, we characterized circulating immune cells using CyTOF on whole blood samples and scRNAseq on PBMCs. We used unbiased clustering on PBMC scRNAseq to identify immune cell populations and compared the relative frequencies across Olink groups. Following this, we integrated our proteomics data with scRNAseq to identify four distinct patterns of immune response in COVID-19. To characterize local changes to the lung immune microenvironment, we obtained BAL samples from COVID^+^, COVID^−^, and convalescent patients and performed scRNAseq. We further expanded our characterization of the lung using MICSSS on lung autopsy samples and quantified changes in myeloid cell infiltration.

### Statistical analysis

Data analysis was performed using Prism version 9.2.0 (GraphPad software) or in R version 4.0.2 and presented as stated in the figure legends. Two-way analysis of variance (ANOVA) with Tukey’s multiple comparisons correction was used for Olink analysis. Normality was determined by quantile-quantile plot. Clinical parameter statistical testing for categorical variables was performed using the chi-square test to determine the overall significance followed by the two-sided Fisher’s exact test between groups. Statistical significance for quantitative clinical parameters was calculated using the Kruskal-Wallis test followed by multiple hypothesis correction with Dunn’s test. Effects of different treatments on Olink cytokine concentrations at different time points were calculated by multiple Welch’s *t* tests with false discovery rate (FDR) correction [a two-stage step-up (Benjamini, Krieger, and Yekutieli) method]. Kruskal-Wallis tests followed by the multiple comparisons test with FDR correction (a two-stage linear step-up procedure of Benjamini, Krieger, and Yekutieli) were used to calculate statistically significant cell frequency changes for CyTOF and scRNAseq. For other cell frequency comparisons with two groups, Mann-Whitney tests were performed. For Spearman correlation coefficient calculations, *P* < 0.05 was considered statistically significant.

## Supplementary Material

Supplementary Figures and Tables

Supplementary Data Files

Reproducibility Checklist

## Data Availability

All data associated with this study are present in the paper or the Supplementary Materials. Clinical data and raw data from patients who consented to data sharing on large public repositories can be found at ImmPort (www.immport.org) under study accession number SDY2011 and at Synapse (www.synapse.org), Synapse ID syn35874390. Code used for MICSSS quantification is available upon request, pending licensing and patent status, by contacting the corresponding author. This work is licensed under a Creative Commons Attribution 4.0 International (CC BY 4.0) license, which permits unrestricted use, distribution, and reproduction in any medium, provided the original work is properly cited. To view a copy of this license, visit http://creativecommons.org/licenses/by/4.0/. This license does not apply to figures/photos/artwork or other content included in the article that is credited to a third party; obtain authorization from the rights holder before using this material.

## The Mount Sinai COVID-19 Biobank Team:

The following investigators and volunteers participated in the Mount Sinai COVID-19 Biobank effort including electronic medical record screening, patient consenting, collection kit assembly, nurse outreach, specimen transport, sample processing, biobanking, data acquisition, data management, and data processing: Charuta Agashe^11^, Priyal Agrawal^11^, Alara Akyatan^11^, Kasey Alesso-Carra^11^, Kimberly Argueta^11^, Eziwoma Alibo^11^, Kelvin Alvarez^11^, Angelo Amabile^11^, Steven Ascolillo^11^, Rasheed Bailey^11^, Craig Batchelor^11^, Priya Begani^11^, Paloma Bravo Correra^11^, Larissa Burka^11^, Sharlene Calarossi^11^, Lena Cambron^11^, Gina Carrara^11^, Serena Chang^11^, Esther Cheng^11^, Jonathan Chien^11^, Mashkura Chowdhury^11^, Grace Chung^11^, Jonathan Chung^11^, Cansu Cimen Bozkus^11^, Phillip Comella^11^, Dana Cosgrove^11^, Francesca Cossarini^11^, Liam Cotter^11^, Arpit Dave^11^, Bheesham Dayal^11^, A. Cassandra De Jesus^11^, Maxime Dhainaut^11^, Rebecca Dornfeld^11^, Katie Dul^11^, Melody Eaton^11^, Nissan Eber^11^, Cordelia Elaiho^11^, Frank Fabris^11^, Jeremiah Faith^11^, Dominique Falci^11^, Susie Feng^11^, Brian Fennessy^11^, Marie Fernandes^11^, Nancy Francoeur^11^, Sandeep Gangadharan^11^, Joanna Grabowska^11^, Gavin Gyimesi^11^, Maha Hamdani^11^, Diana Handler^11^, Jocelyn Harris^11^, Matthew Hartnett^11^, Sandra Hatem^11^, Manon Herbinet^11^, Elva Herrera^11^, Arielle Hochman^11^, Gabriel E. Hofman^11^, Laila Horta^11^, Etienne Humblin^11^, Jessica S. Johnson^11^, Gurpawan Kang^11^, Neha Karekar^11^, Subha Karim^11^, Geoffrey Kelly^11^, Jessica Kim^11^, Jose Lacunza^11^, Alana Lansky^11^, Dannielle Lebovitch^11^, Grace Lee^11^, Gyu Ho Lee^11^, Jacky Lee^11^, John Leech^11^, Lauren Lepow^11^, Mike Leventhal^11^, Lora E. Liharska^11^, Katherine Lindblad^11^, Alexandra Livanos^11^, Rosalie Machado^11^, Zafar Mahmood^11^, Kelcey Mar^11^, Shrisha Maskey^11^, Paul Matthews^11^, Katherine Meckel^11^, Saurabh Mehandru^11^, Anthony Mendoza^11^, Cynthia Mercedes^11^, Dara Meyer^11^, Ronaldo Miguel de Real^11^, Gurkan Mollaoglu^11^, Sarah Morris^11^, Emily Moya^11^, Nicole Ng^11^, Marjorie Nisenholtz^11^, George Ofori-Amanfo^11^, Kenan Onel^11^, Merouane Ounadjela^11^, Manishkumar Patel^11^, Vishwendra Patel^11^, Cassandra Pruitt^11^, Shivani Rathi^11^, Jamie Redes^11^, Ivan Reyes-Torres^11^, Alcina Rodrigues^11^, Alfonso Rodriguez^11^, Vladimir Roudko^11^, Evelyn Ruiz^11^, Kevin Rurak^11^, Pearl Scalzo^11^, Ieisha Scott^11^, Pedro Silva^11^, Alessandra Soares Schanoski^11^, Hiyab Stefanos^11^, Meghan Straw^11^, Sayahi Suthakaran^11^, Collin Teague^11^, Kevin Tuballes^11^, Bhaskar Upadhyaya^11^, Verena Van Der Heide^11^, Natalie Vaninov^11^, Daniel Wacker^11^, Laura Walker^11^, Hadley Walsh^11^, C. Matthias Wilk^11^, Lillian Wilkins^11^, Jessica Wilson^11^, Karen M. Wilson^11^, Hui Xie^11^, Luyi Xu^11^, Li Xue^11^, Naa-akomaah Yeboah^11^, Nancy Yi^11^, Mahlet Yishak^11^, Sabina Young^11^, Alex Yu^11^, Nicholas Zaki^11^, Nina Zaks^11^, and Renyuan Zha^11^.
