# Supplementary material for: A shift in lung macrophage composition is associated with COVID-19 severity and recovery
Source: Sci Transl Med. Author manuscript; Available in PMC 2023 Apr 20. (PMC10117220; doi:10.1126/scitranslmed.abn5168)
Supplement: Supplementary Figures and Tables [file NIHMS1890556-supplement-Supplementary_Figures_and_Tables.pdf]

Science Translational Medicine
AAAS

Supplementary Materials for

# A shift in lung macrophage composition is associated with COVID-19 severity and recovery

Steven T. Chen *et al.*

Corresponding author: Miriam Merad, miriam.merad@mssm.edu

*Sci. Transl. Med.* **14**, eabn5168 (2022)
DOI: 10.1126/scitranslmed.abn5168

**The PDF file includes:**

Materials and Methods
Figs. S1 to S9
Tables S1 to S4
Legends for data files S1 to S6

**Other Supplementary Material for this manuscript includes the following:**

Data files S1 to S6
MDAR Reproducibility Checklist

## MATERIALS AND METHODS

### Mount Sinai COVID-19 Biobank and Ethics Statement

Electronic medical records (EMR) from patients admitted to the Mount Sinai Hospital for suspected or confirmed COVID-19 were screened each morning by a team of volunteers and physicians for enrollment into the Mount Sinai coronavirus disease 2019 (COVID-19) Biobank. Due to the difficulty of obtaining informed consent from patients during the pandemic, the Institutional Review Board (IRB) approved sample collection from patients before consent was obtained. Sample collection occurred at the same time as clinical biospecimen collection and included at most an extra 5 to 10 cc of blood. Patients were made aware of planned sample collection with documents provided during hospital registration and provided instructions for opting out. Limited existing clinical data were obtained from the medical record and associated with research samples. Patient consent, including consent for genetic profiling for research and data sharing, was subsequently obtained by contacting patients by hospital-room phone, phone call after discharge, or through legally authorized representatives after death of patients. As research sample laboratory processing needed to begin urgently after sample collection, a portion of the data were generated prior to obtaining informed consent. COVID$^-$ serum, whole blood, and peripheral blood mononuclear cell (PBMC) samples were obtained from consented COVID$^-$ volunteers of the Mount Sinai community. This study was approved by the Institutional Review Board of the Mount Sinai School of Medicine under STUDY-20-03276. Per STUDY-20-03276 guidelines, in circumstances where consent was unable to be obtained (11.6% of subjects, 0% of subjects who completed the post-discharge checklist), data already generated could continue to be used for analysis purposes only when not doing so would have compromised the scientific integrity of the work. Data have been made available on ImmPort (SDY2011) for patients who consented to data sharing on large public repositories. EMR and deidentified clinical data for each patient were pulled from Epic electronic health record using Epic Hyperspace, Epic Clarity, and the Mount Sinai Data Warehouse, and summarized per 24 hour period measures throughout length of hospital stay. Period window was defined as 12:00 PM to 12:00 PM to match blood draw time.

### Clinical Blood Sample Collection and Processing

At each collection timepoint, COVID$^+$ patient or COVID$^-$ volunteer serum was collected in a BD Vacutainer Plus Plastic Blood Collection Tubes with Polymer Gel for Serum Separation tube (SST). Two BD Vacutainer cell preparation tube (CPT) with Sodium Heparin were collected for Plasma and PBMC collection. Samples were obtained by nurses or phlebotomists as part of clinical care, collected from hospital floors by "Running Team" volunteers, and delivered to the laboratory for processing. Blood samples were kept on gentle agitation at room temperature (RT) and processed by Blood Processing Team volunteers of the Mount Sinai COVID-19 Biobank in Biosafety level 2 plus (BSL2$^+$) facilities on the day of collection.

SST tubes were centrifuged at RT at 1300 relative centrifugal force (rcf) for 10 minutes (mins) and serum was banked into cryovials for storage in liquid nitrogen. COVID$^+$ patient or COVID$^-$ volunteer whole blood (WB) samples for cytometry by time of flight (CyTOF) were taken from CPT tubes and directly stained with a lyophilized antibody panel using Fluidigm MaxPar Direct Immune Profiling Assay (MDIPA) tubes for 30 mins at RT. Stained WB samples were then stabilized and fixed with Prot1 Proteomic Stabilizer for 10 mins at RT before storage at -80°C as previously described*(45)*. CPT tubes were then centrifuged at 1800 rcf for 15 mins to separate plasma and PBMC. Plasma was aspirated and banked into cryo-vials for storage in liquid nitrogen. The PBMC cell layer was collected, washed with phosphate buffered saline (PBS) and collected by

centrifugation at 300 rcf for 15 mins. Cell viability and counts were assessed by acridine orange and propidium iodide (AOPI) staining in automated Nexcelom Cellometer Cell Counters. PBMC were resuspended at a concentration of ~10x$10^6$ cells/mL in Human Serum and 10% dimethyl sulfoxide (DMSO) and stored at -80°C for 24 hours before transfer to liquid nitrogen storage.

**Olink measurements of COVID-19 serum, data normalization, and clustering analysis**

Olink was performed on $COVID^-$ volunteer and $COVID^+$ patient serum samples in $BSL2^+$ according to manufacturer instructions. Count (Ct) values were generated by Olink NPX manager software. To control for technical variability between plates, we included in each plate 2 technical control replicates from a single mixture of pooled blood from healthy donors and estimated a control value per plate, defined as:

$$Ctrl^a(plate_i) = \frac{1}{|j \in T|\ plate_j = plate_i|} \sum_{j \in T|\ plate_j = plate_i} log\left(Ct_j^a\right)$$

Where: a is a given analyte, i is a given sample, T is the set of technical control replicates, $plate_i$ is the plate of sample i, and $Ct_s^a$ is the raw Olink Ct value of analyte a in sample s. Normalized Ct values $Z(Ct'^a_s)$ were defined as the z-scores of the plate-adjusted, log transformed Ct values $Ct'^a_s$:

$$Ct'^a_s = log\left(Ct_i^a\right) - Ctrl^a_{plate(i)}$$

Samples with similar normalized Ct profiles $Z(Ct'^a_s$ were clustered using Kmeans++ (https://github.com/tanaylab/tglkmeans) with k=15. Effects of different treatments on Olink cytokine concentrations at different timepoints were calculated by Welch's t-tests with false discovery rate (FDR) correction. Olink protein module scores were calculated by averaging the normalized z-scores of each module analyte. Univariate logistic regression was performed using R package glmnet_4.1. Area under the curve (AUC) values were calculated using R package pROC_1.16.2. Pearson correlation coefficients of Olink clusters were calculated using the averaged values of each Olink analyte per cluster.

**CyTOF Data acquisition and analysis**

MDIPA stained WB samples were thawed using the SmartTube Prot 1 Thaw/Erythrocyte Lysis protocol. Samples were subsequently barcoded and pooled utilizing the Fluidigm Cell-ID 20-Plex Pd Barcoding Kit and stained with an antibody cocktail against fixation stable markers for more in depth immune profiling. Following sample barcoding and staining, samples were fixed with 2.4% paraformaldehyde in PBS with 0.08% saponin and 125 nM Iridium (Ir) for 30 mins at RT and stored in Cell Staining Buffer until acquisition. Immediately prior to data acquisition, samples were washed once in Cell Acquisition Solution and resuspended at a concentration of 1x10^6 cells/mL for acquisition (including 10% Fluidigm EQ Normalization Beads). The resuspended cells were then acquired on the Helios Mass Cytometer supplemented with a wide bore injector at an event rate below 400 events/second. After data acquisition, samples were de-barcoded using the Astrolabe Diagnostics platform. Cell populations were identified by a combination of an automated approach using the Astrolabe and manual gating as previously described*(45)*.

**PBMC preparation for scRNAseq**

$COVID^+$ patient and $COVID^-$ volunteer PBMC samples were selected based on manual EMR chart review taking into account, and controlling for patient demographics and treatments. Frozen PBMC were thawed at 37°C and resuspended in RPMI-1640 media+ 10% fetal bovine serum (FBS) with 25 U/mL Benzonase before centrifugation at 350 rcf for 5 mins. Cells were resuspended in media and viable cells were counted by AOPI staining in Nexcelom Cellometer

Cell Counters. Combinatorial hashes were prepared in wash buffer (PBS + 0.5% bovine serum albumin (BSA)). 500,000 live cells were stained with hashes for 20 mins on ice before 3 washes in wash buffer. Cells were filtered through a 70 μm filter and then a 40 μm filter twice. Filtered cells were counted and loaded with a targeted cell recover of 35,000 cells/lane across 8 lanes of 5' v1.1 NextGEM assay. PBMC samples were processed, hashed, and sequenced in 3 separate batches.

### Patient selection for bronchoalveolar lavage

Respiratory samples for research were allocated from BAL obtained from patients ≧ 18 years of age undergoing bronchoscopy with BAL fluid collection for clinical reasons. Patient groups included the following: (1) positive SARS-CoV-2 polymerase chain reaction (PCR) with COVID-19 related acute respiratory failure requiring intubation and mechanical ventilation; and (2) negative SARS-CoV-2 PCR with suspected lung cancer. Patients in Group 2 who had previous positive SARS-CoV-2 PCR or SARS-CoV-2 antibodies were designated as COVID-19 convalescent. Informed consent for bronchoscopy with BAL fluid collection was obtained separately from consent for research.

For Group 1, patients were identified by the attending critical care physician providing clinical care. Patients were intubated at the discretion of the critical care team for progressive respiratory failure, as evident by worsening hypoxemia, hypercapnia, or work of breathing despite support by high-flow nasal cannula oxygen or non-invasive ventilation. Bronchoscopy with BAL fluid collection was performed within 72 hours of first intubation if clinically indicated. At our center, all patients with COVID-19, including those with COVID-19-related respiratory failure, were managed according to guidelines developed and updated by the Mount Sinai Health System as new data regarding care of patients with COVID-19 became available*(46)*. Patients requiring intubation and mechanical ventilation for COVID-19-related respiratory failure were additionally managed with a low tidal volume ventilation strategy*(47)*.

For Group 2, patients were identified by the pulmonologist and physician assistant who performed the bronchoscopy with BAL fluid collection, which was carried out for suspected lung cancer requiring a diagnostic and staging procedure. Negative SARS-CoV-2 PCR was obtained 2 to 5 days prior to bronchoscopy. Patients were selected on the basis of suspected lung cancer not greater than 5 cm in greatest dimension. Patients with known history of, or clinical suspicion for, active infectious or inflammatory lung diseases were excluded.

### BAL fluid collection

All respiratory specimens were collected using sterile, flexible, fiberoptic bronchoscopes. Bronchoscopes were flushed prior to the procedure with 5 mL sterile saline, which was collected for research. For Group 1, a single-use bronchoscope was inserted through the endotracheal tube. For Group 2, a reusable bronchoscope was inserted through a laryngeal mask airway or endotracheal tube placed for the procedure. After airway inspection, the bronchoscope was wedged in a distal airway of interest selected by pre-procedure imaging. Sterile saline was instilled in 30 mL aliquots (up to 90 and 210 mL for Groups 1 and 2, respectively) and aspirated. Aspirated BAL fluid was split into parts for clinical use, which included fluid sent for clinical microbiological analysis, and research use, which was transported immediately to the research laboratory on ice and processed as below.

### BAL fluid processing

BAL samples were processed within 30 mins of sample collection. Collected BAL was filtered twice through 70 μM filters and centrifuged at 350 rcf for 5 mins at 4°C. BAL supernatant was collected and treated with 0.1% Triton X-100 for 1 hour to inactivate virus before aliquoting into cryovials for storage at -80°C. BAL cells were incubated with Red Blood Cell Lysis buffer (Thermo Fisher Scientific) for 5 mins at RT before washing with PBS+ 0.5% BSA and centrifugation at 350 rcf for 5 mins at 4°C. Viable cells were counted by AOPI staining in Nexcelom Cellometer Cell Counters. Two lanes of 8000 cells from each sample were loaded onto the 10x Chromium Controller for scRNAseq, 5' v1.1 NextGEM assay.

**PBMC and BAL scRNAseq data processing**

For all scRNAseq datasets, debris and empty droplets were identified with cells that had gene expression (GEX) UMI counts<22. Cells were identified by finding local minimum in the GEX UMI distribution, keeping only cells ≧ the local minimum UMI count. On average, this was 371 UMI counts. For PBMC scRNAseq, hashtag oligo (HTO) UMI counts <22 and excluded from further analysis. For HTO transformation, each feature in the HTO matrix (linear space) was subtracted from its $5^{th}$ quantile and divided by its $95^{th}$ quantile. Each cell was subsequently divided by its UMI sum. Because hashing reads were not consistently detected, we underwent additional processing to dehash cells.

For initial mapping of cells to samples using only HTOs, a "key" matrix with biological samples as rows and HTO features as columns was created. Each unit in the matrix was populated with a value of "1" if the sample was supposed to be positive for the hashtag and "0" if it was not. For each cell, pairwise distances with a cosine similarity metric were computed from the key matrix to generate a cosine similarity matrix. Samples with the highest cosine similarity could then be assigned to the cell. Initially assigned mappings were called "hto-ini".

Additionally, a signal to noise ratio (SN) was for each cell was calculated by subtracting the highest cosine similarity from the second highest cosine similarity. The SN ratios for each initially assigned sample usually followed a bimodal distribution. The relative minima of the parametric density of SN ratios was used in order identify the local minimum of this distribution. Only cells with SN ≧ the local minimum were kept as the final cells belonging to that sample. We call these final mappings "sample-hto"

We used Souporcell to cluster the cells based on the polymorphisms detected from the RNA-Seq alignment*(48)*. We also inputted the genotypes inferred from whole genome DNA-Seq data as a reference in Souporcell in order to map the clusters to respective patients. Next, we leveraged the Souporcell subject mappings along with the initially assigned HTO mappings (hto-ini) in order to deconvolute the patient to the various timepoints. For patients with single timepoints, we assigned the entire Souporcell cluster to the sample. For patients with multiple timepoints, we performed the hashtag de-multiplexing strategy described above to map the cells from the cluster to the patient's timepoints. We called these mapped cells "sample-soc-hto". For final mappings, we intersected the output from both strategies above ("sample-hto" and "sample-soc-hto") in order to get a consensus cell-sample mapping. Only these consensus cells were used for further downstream analysis.

**scRNAseq Analysis**

Briefly, mRNA reads were tagged with a cell barcode and UMI. These reads were aligned, and count matrices were built. Cell barcodes with at least 500 UMIs were extracted, and cells that were comprised of more than 25% of reads from the mitochondrial genome were filtered from

subsequent analysis. The variability in cell counts or UMI counts across samples were not confounding variables in downstream analyses. To account for controlled sampling of each patient sample, each sample was downsampled to 250 cells. The Seurat R package was then used for scaling, batch-aware integration, clustering, dimensionality reduction, and downstream differential gene expression analyses *(49–52)*. To adjust for batch effects, anchors, defined as overlapping shared nearest neighbors, were imputed and used to transform all datasets into a complete shared space. The function SCTransform was used to scale and identify variable genes that constituted principal components for principal component analysis (PCA). The first 15 principal components were used to perform UMAP reduction, once a shared nearest-neighbor graph had been generated and clustering had been performed based on the Louvain method for community detection. To identify the cell clusters, cells were down-sampled to 2000 UMIs per cell and variable genes were selected. Gene module analysis was performed by computing a Pearson correlation matrix between genes for each sample using the scDissector R package and grouping highly correlated genes into gene modules by hierarchical clustering*(53)*. Based on this, cell types were manually annotated. Chi-square analysis of gene expression was done across all immune cell clusters, then on specific subsets of lineages, identified subsets of cell states within the myeloid and lymphoid lineages and major cell types.

scRNAseq immune cell cluster frequency correlations, and integrated scRNAseq cell frequencies and Olink proteomics were calculated using the corrplot package (v0.88) and visualized using the pheatmap package (v1.0.12) in R. To identify differentially expressed genes between alveolar macrophages (AM), we computed relative fold change in gene expression between groups and plotted these values with respect to gene expression (total UMI for a gene of interest), represented as a fraction of all UMI per cell. Significant differentially expressed genes were determined using the *FindMarkers* function from the Seurat R package with default parameters and selecting for genes with an adjusted p-value of less than 0.05.

**Lung Autopsy Tissue Section Preparation**

Lung autopsy samples were collected within 24 hours of death (average 10.1±6.2 hours) and fixed in 10% neutral-buffered formalin for 24 hours before transfer to 70% Ethanol (EtOH). Samples were then embedded in paraffin and 4 μM tissue sections formalin fixed paraffin embedded (FFPE) sections were cut onto glass slides and baked at 37ºC overnight.

Using an Autostainer (Bond Rx, Leica Biosystems), slides were covered with covertiles (Bond Universal Covertiles, Leica biosystems) and baked for 10 mins at 57ºC. Slides were deparaffinizied in dewax solution and rehydrated in decreasing concentrations of EtOH. Tissue sections were then incubated in antigen retrieval solution (pH 6 or 9) at 95ºC for 20 mins. Tissue sections were incubated in 3% hydrogen peroxide (Bond Polymer Refine Detection Kit DS9800, Leica Biosystems) for 15 mins to block endogenous peroxidases. Next, tissue sections were incubated in serum-free protein block solution (Dako, X0909) for 30 mins to block nonspecific antibody binding. After the first staining cycle, Fab fragments (AffiniPure Fab Fragment Donkey anti-mouse (715-007-003) or anti-rabbit IgG (711-007-003) against that primary antibody species were used to block carry-over staining whenever there was a repeat of same primary antibody species. Primary antibody staining was performed for 1 hour at RT or overnight at 4°C followed by secondary antibody staining. For primary antibody staining, anti-human CD14 (Sigma-Aldrich, AMAB90897) was used at a dilution of 1:1000; anti-human CD68 (Dako, M0814) was used at a dilution of 1:100; anti-human S100A12 (Atlas Antibodies, HPA002881) was used at a dilution of 1:2500; anti-human FABP4 (R&D Systems, AF3150) was used at a dilution of 1:70; anti-human CD66b (BD Pharmingen, 555723) was used at a dilution of 1:600, anti-human Foxp3 (Abcam, ab20034) was used at a dilution of 1:80, anti-human CD3 (Ventana, 90-4341), was used pre-

diluted as provided by the supplier; anti-human CD8 (Dako, M7103) was used at a dilution of 1:100; anti-human CD20 (Dako, M0755) was used at a dilution of 1:250. Anti-mouse (Dako K4001), anti-rabbit (Dako K4003), and anti-goat (R&D Systems VC004) secondary antibodies were used pre-diluted as provided by the supplier. Polymer detection system (Bond Polymer Refine Detection Kit, DS9800, Leica Biosystems) was used for horseradish peroxidase signal amplification. Chromogenic revelation was performed using ImmPact AEC (3-amino99-ethylcarbazole) substrate (Vector Laboratories, SK4205) for preset incubation times. Slides were counterstained with hematoxylin (Bond Polymer Refine Detection Kit, DS9800, Leica Biosystems).

For manual staining, slides were mounted with a glycerol-based mounting medium (Dako, C0563) and scanned for digital imaging (Hamamatsu NanoZoomer S60 Whole Slide Scanner). The same slides were successively stained, as per MICSSS protocol. Coverslips were removed by placing slides in a rack and immersing in hot tap water at 56ºC until mounting media dissolved. Chemical destaining between stains was performed by immersing slides in gradually diluted EtOH solutions.

**MICSSS co-expression analysis**

To analyze marker coexpression, a pseudofluorescence composite image of all chromogenic markers was created. The same region of interest (ROI) were selected from images of each marker in QuPath (https://qupath.github.io/). and exported as PNG formatted images without downsampling**.** Images of different immunostains belonging to the same ROI were transferred to Fiji-ImageJ and co-registered using the TrakEM2 plug-in*(54, 55)*. Color deconvolution was performed using H-AEC vectors for each image to split the RGB images into three 8-bit channels including hematoxylin (blue), AEC (red chromogen color), and residual (green) channel. The best hematoxylin channel was selected as the nuclear channel. AEC channels representing staining of each marker were assigned to different colors by using the lookup tables (LUTs) function of Fiji and hematoxylin channel was assigned to blue color to mimic fluorescent DAPI staining. Next, color inversion was done on all channels and then merged to achieve a multiplexed pseudofluorescent image. We optimized brightness and contrast settings to facilitate visualization for each immunostain channel by comparison with original chromogen images but did not change underlying image pixel values for quantification.

**MICSSS quantification**

Stained images were scanned at 40x resolution into the .ndpi format and uploaded to Amazon Web Services (AWS) super computer clusters for high-speed analysis on a Python-based Anaconda Jupyter Notebook. Raw red-green-blue (RGB) thumbnail 1.25x resolution images were analyzed using an in-house tissue recognition algorithm that enhanced tissue contours and used optical densities of pooled pixels across the image to determine a tissue mask. The image was then rigidly registered with the corresponding images across all markers for the same tissue within the MICSSS panel. This registration used a third party SimpleElastic package for Python (https://simpleelastix.readthedocs.io/RigidRegistration.html). Following linear registration, the highest resolution image for each marker (40x) was spliced into multiple tiles that spanned approximately 2000 µm in each dimension with 20% surface area overlap in each direction. Each tile was then denoted with an X,Y pooled pixel coordinate value so that the appropriate corresponding tiles across all the markers in the panel for the same tissue would be analyzed together. Each set of tiles was deconvoluted to extract the hemoxylin channel, which remained consistent across all markers. The hematoxylin channel was then registered with an affine registration (which accounts for shear, scale, rotation, and translational dislocation) and a "b-

spline" elastic warping to account for any local tissue warping or tissue damage (https://simpleelastix.readthedocs.io/NonRigidRegistration.html)

The vector field transformation matrix produced from the high-resolution affine and b-spline registrations was then applied to the raw RGB tile. Registered RGB tiles were analyzed in parallel across the multiple cores of the AWS supercomputer, trimmed to eliminate overlap, and concatenated to produce one final elastically registered RGB image per marker. 100 ROIs of about 500x500 μm were randomly chosen in the image based on where tissue resided. These were chosen from the last tissue mask in the panel to account for any tissue damage or warping. Each of these ROI was processed in parallel across the multiple cores in the AWS supercomputer. Next, we used the Stardist package for Python (https://github.com/stardist/stardist) that was trained with hematoxylin and eosin staining, to segment each ROI, and to determine the centroids and morphological properties of each determined cell. We previously optimized the sensitivity of this algorithm with these tissues and therefore used an overall sensitivity value of 0.1 for the algorithm. This algorithm provides information for the nucleus of each cell in the ROI, which we then artificially expanded by 5 μm to simulate the cell's cytoplasm. Overlapping cytoplasm from adjacent cells in dense regions were condensed to prevent overcounting surface area.

Each cell per ROI was then analyzed for marker positivity. ROI were deconvoluted for its AEC detection channel and each cell was translated to a median AEC value from pixels that resided in its nucleus and artificially expanded cytoplasm. If the median AEC value for the cell was above the threshold value for positivity deemed for that marker, the cell was considered positive. Threshold values per marker were determined with a pathologist. The percentage of positive cells was determined by the number of cells above the threshold for that marker over the total number of cells in that ROI. Co-expression analysis was performed by determining if a cell was positive for the multiple markers of interest over the total number of cells in that ROI. We plotted each value per ROI.

## SUPPLEMENTARY FIGURES

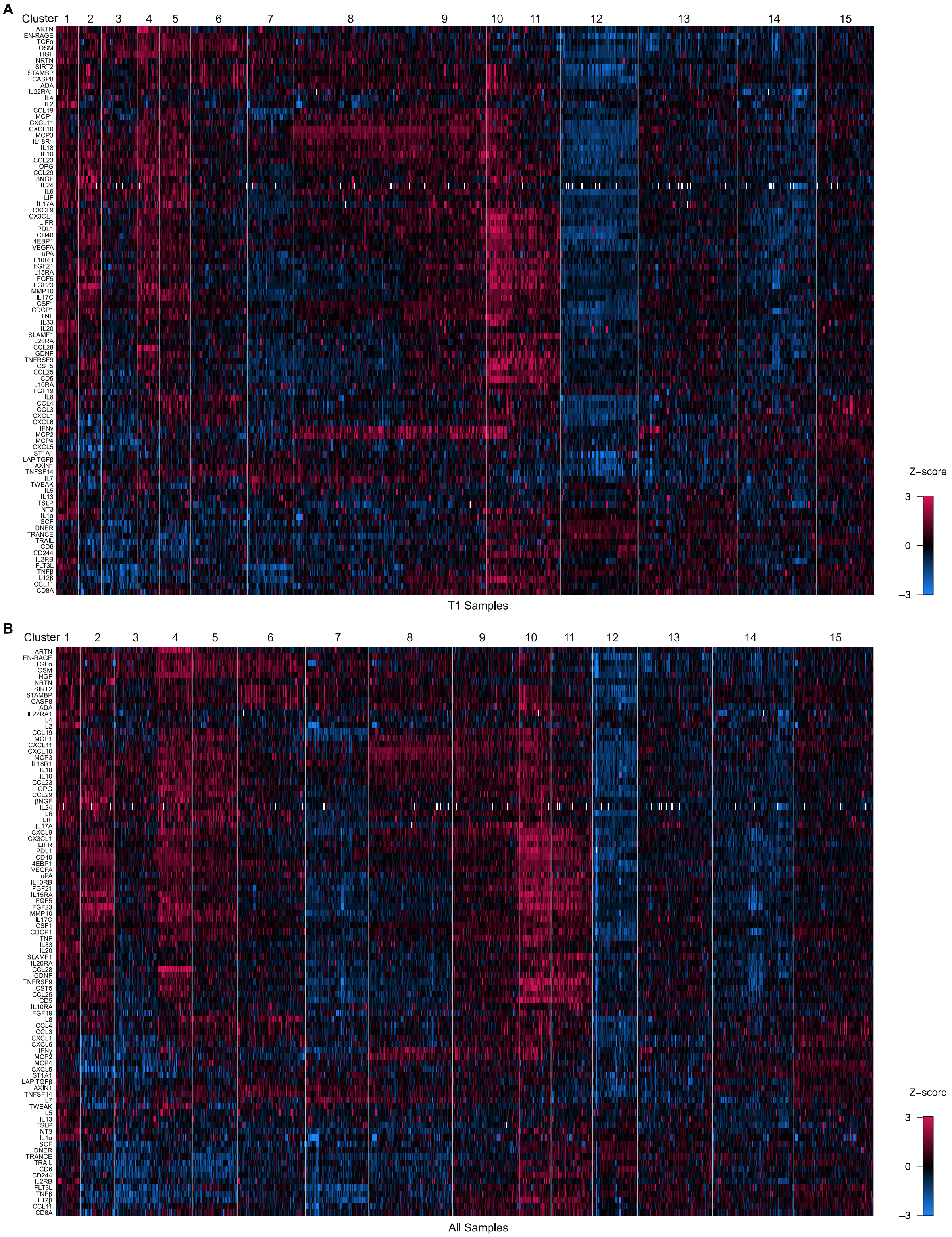
A
Cluster 1 2 3 4 5 6 7 8 9 10 11 12 13 14 15
T1 Samples
Z-score
3
0
-3
B
Cluster 1 2 3 4 5 6 7 8 9 10 11 12 13 14 15
All Samples
Z-score
3
0
-3

**Supplementary Figure 1. Unsupervised K-means++ clustering of COVID-19 serum proteomics identified 15 distinct immune patterns. (A)** The heatmap shows cytokine profiles of serum samples from $COVID^+$ patients and $COVID^-$ volunteers measured with the Olink inflammation panel. Unsupervised K-means++ clustering was performed on normalized values (z-scores) for timepoint 1 (T1) serum samples (*n=598*). 15 clusters were identified. The rows denote each protein measured and the columns denote proteomic profile of each patient serum sample. **(B)** The heatmap shows cytokine profiles of serum samples from $COVID^+$ patients and $COVID^-$ volunteers measured with the Olink inflammation panel. Unsupervised K-means++ clustering was performed on normalized values (z-scores) for all serum samples across all timepoints (*n=2001*). 15 clusters were identified. The rows denote each protein measured and the columns denote proteomic profile of each patient serum sample.

A

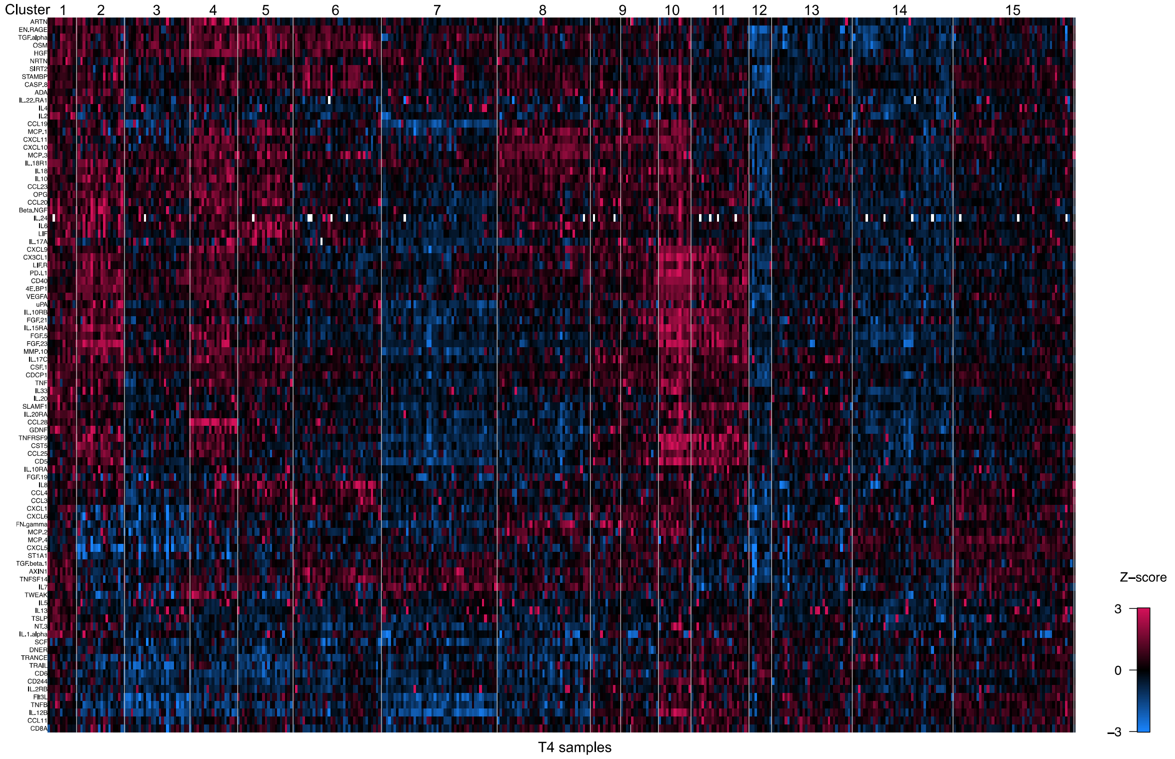
Cluster
1 2 3 4 5 6 7 8 9 10 11 12 13 14 15
T4 samples
Z-score
3
0
−3

B

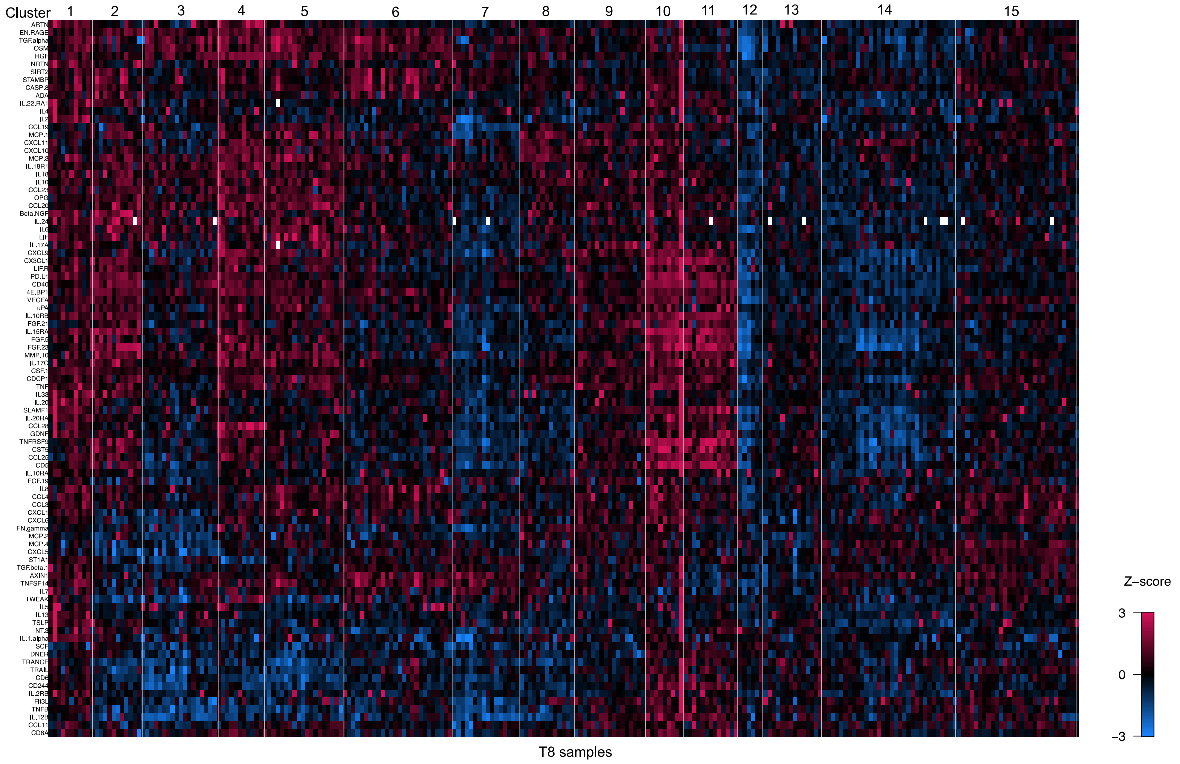
Cluster
1 2 3 4 5 6 7 8 9 10 11 12 13 14 15
T8 samples
Z-score
3
0
−3

**Supplementary Figure 2. Unsupervised K-means++ clustering of T4 and T8 serum samples. (A)** The heatmap shows cytokine profiles of serum samples from COVID$^+$ patients measured with the Olink inflammation panel. Unsupervised K-means++ clustering was performed on normalized values (z-scores) for timepoint 4 (T4) serum samples (*n=377*). The rows denote each protein measured and the columns denote proteomic profile of each patient serum sample. **(B)** An averaged z-scored heatmap showing cytokine profiles of sera from COVID$^+$ patients measured with the Olink inflammation panel. Unsupervised K-means++ clustering was performed on normalized values (z-scores) for timepoint 8 (T8) serum samples (*n=234*). The rows denote each protein measured and the columns denote proteomic profile of each patient serum sample.

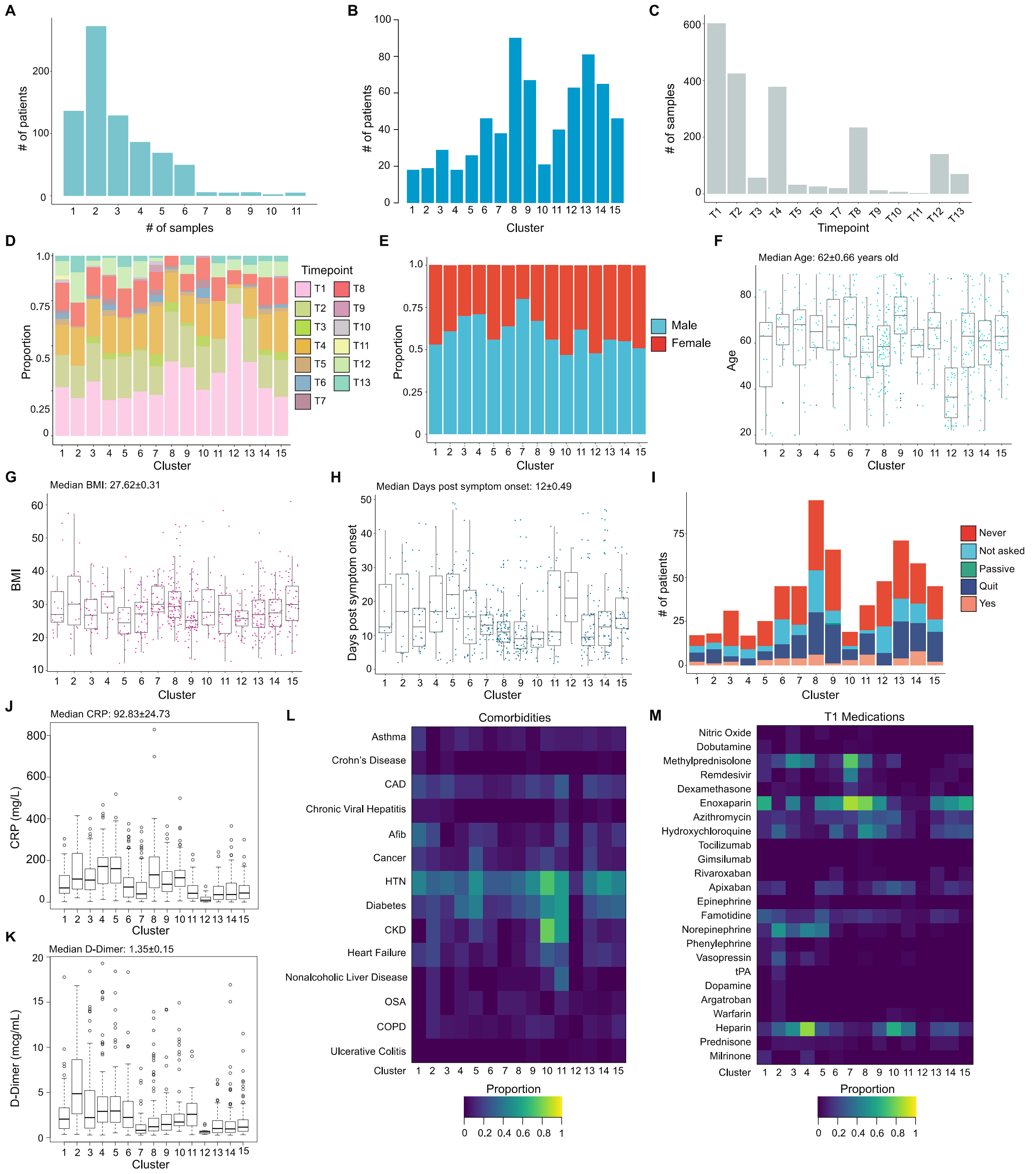


**Supplementary Figure 3. Clinical characteristics of Olink serum clustering analysis. (A)** The histogram shows the number of patients by number of serum samples analyzed is shown (*n=583*). **(B)** The histogram shows the number of COVID$^{+}$ patients or COVID$^{-}$ volunteers per Olink cluster is shown (*n=628*). **(C)** The histogram shows the number of serum samples acquired for each timepoint is shown (*n=2001*). **(D)** Timepoint distribution of serum samples is shown per Olink cluster (*n=2001*). **(E)** Proportion of patient sex is shown based on Olink cluster (*n=628*). (**F to H**)

The boxplots shows all biobank participants' age **(F)**, body mass index (BMI) **(G)** (*n=629*), and days post symptom onset at the time of first sampling for COVID$^+$ patients (*n=583*) **(H)** across Olink clusters. Cluster assignments were set by the first timepoint for each individual **(I)** The stacked histogram shows smoking status for COVID$^+$ patients by Olink Cluster (*n=583*). (**J and K**) Boxplots show C-reactive protein (CRP) **(J)** or D-Dimer concentrations **(K)** for the first available COVID$^+$ patient samples (*n=583*). Cluster assignments were set by the first timepoint for each patient. **(L)** The heatmap shows the proportion of COVID$^+$ patients with comorbidities by Olink Cluster. Cluster assignments were set by the first timepoint for each patient (*n=583*). **(M)** The heatmap shows the proportion of COVID$^+$ patients receiving medications at the time of first sampling (*n=583*). For box plots, each dot represents a patient sample; the center line indicates median; box limits indicate 25$^{th}$ and 75$^{th}$ percentile; whiskers indicate 1.5x interquartile range. CAD, coronary artery disease; Afib, atrial fibrillation; HTN, hypertension; CKD, chronic kidney disease; OSA, obstructive sleep apnea; COPD, chronic pulmonary obstructive disease.

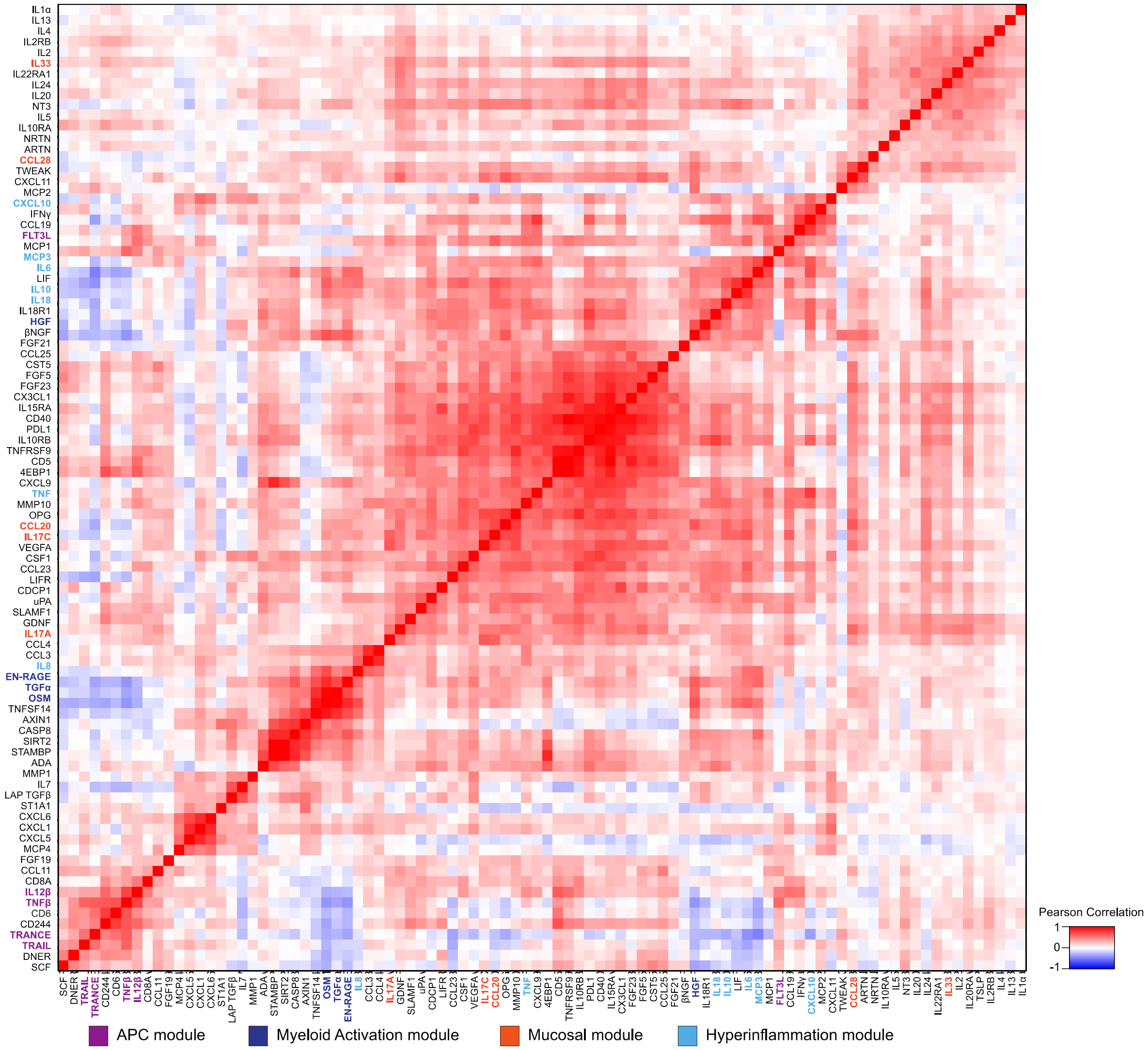


**Supplementary Figure 4. Covariance matrix of Olink analytes in COVID-19 serum.** Heatmap showing the Pearson correlations between Olink analytes for all COVID$^{+}$ patient and COVID$^{-}$ volunteer serum samples (*n=2001*). Olink analytes for protein modules are highlighted.

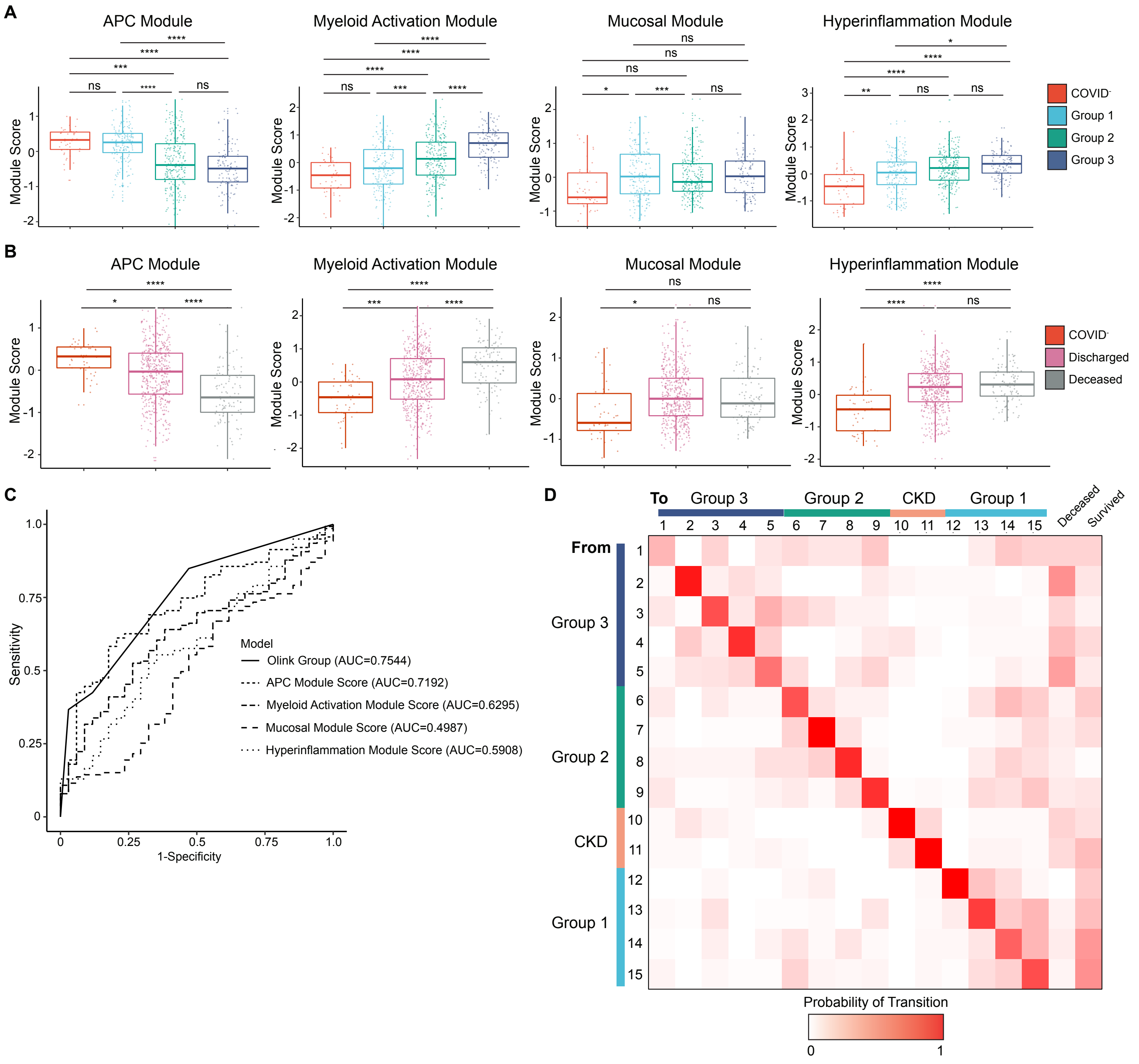

A
APC Module
Myeloid Activation Module
Mucosal Module
Hyperinflammation Module
Module Score
COVID-
Group 1
Group 2
Group 3
B
APC Module
Myeloid Activation Module
Mucosal Module
Hyperinflammation Module
Module Score
COVID-
Discharged
Deceased
C
Sensitivity
1-Specificity
Model
Olink Group (AUC=0.7544)
APC Module Score (AUC=0.7192)
Myeloid Activation Module Score (AUC=0.6295)
Mucosal Module Score (AUC=0.4987)
Hyperinflammation Module Score (AUC=0.5908)
D
To
From
Group 3
Group 2
CKD
Group 1
Deceased
Survived
Probability of Transition
0
1

**Supplementary Figure 5. Olink protein module scores are stable and associated with clinical outcome. (A)** The boxplots show Olink module score comparisons of first available serum samples by Olink group (*n=628*). **(B)** The boxplots show Olink module score comparisons of first available serum samples by patients' final clinical outcome (*n=628*). **(C)** Univariate logistic analysis and AUC of Olink group assignment and protein module scores of first available patient serum samples for prediction of survival is shown. **(D)** The heatmap shows discrete time Markov chain analysis probability of transition between all $COVID^+$ serum samples (*n=2001*) by Olink clusters and clinical outcome. For box plots, each dot represents a patient sample; the center line indicates median; box limits indicate $25^{th}$ and $75^{th}$ percentile; whiskers indicate 1.5x interquartile range. $COVID^-$ samples were obtained from healthy volunteers (A, B, D). Statistical significance in (A and B) was determined by two-way ANOVA with Tukey's Multiple Comparisons correction. ns, not significant; *adj. p<0.05; ***adj. p<0.001; ****adj. p<0.0001.

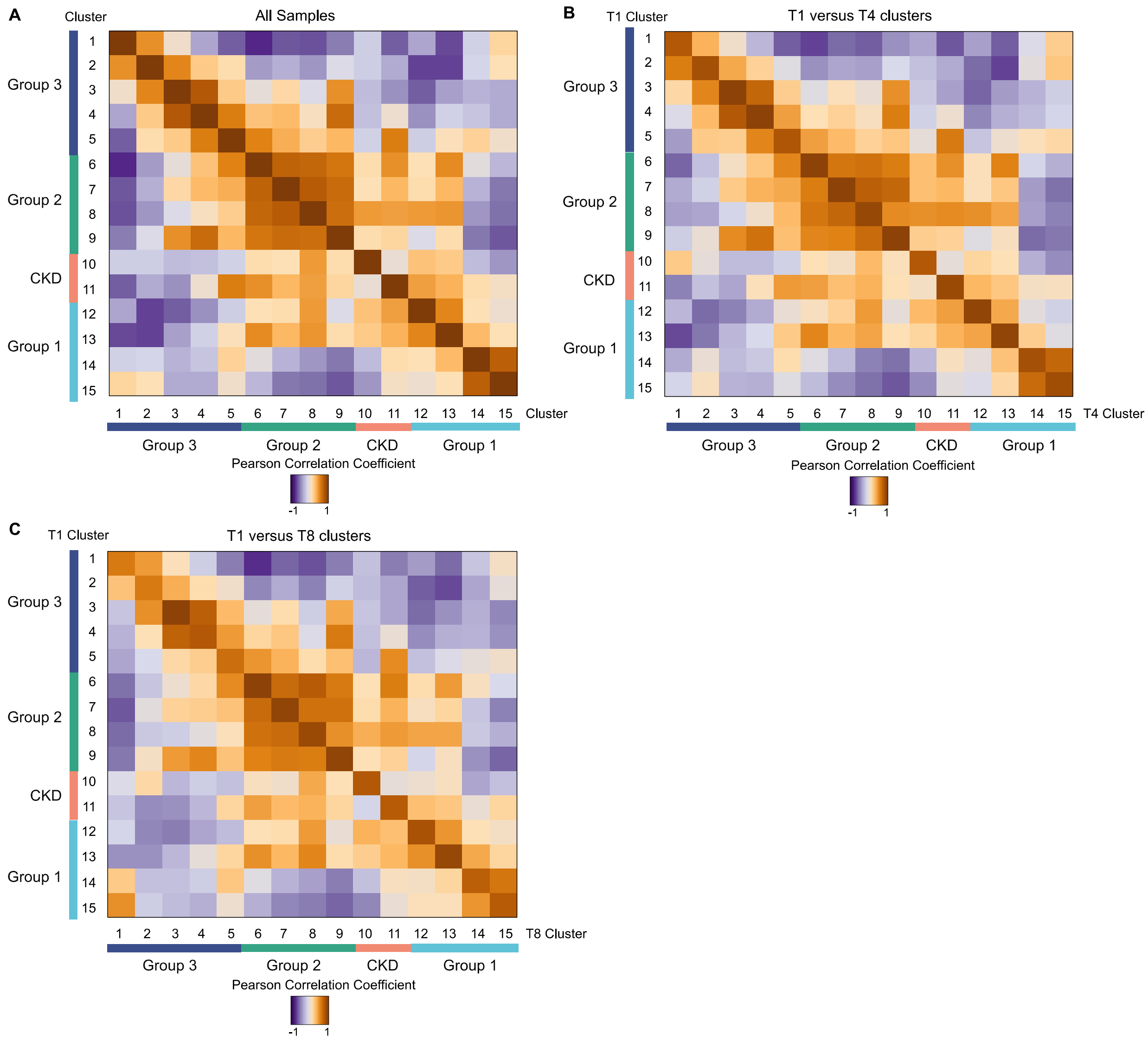


**Supplementary Figure 6. Olink cytokine clusters are strongly correlated by group and across timepoints (A)** The heatmap shows Pearson correlation coefficients of averaged cytokine concentrations by Olink cluster for all COVID$^+$ and COVID$^-$ serum samples. **(B)** The heatmap shows Pearson correlation coefficients of averaged cytokine concentrations between T1 and T4 COVID$^+$ serum samples organized by Olink cluster. **(C)** The heatmap shows the Pearson correlation coefficients of averaged cytokine concentrations between T1 and T8 COVID$^+$ serum samples organized by Olink cluster.

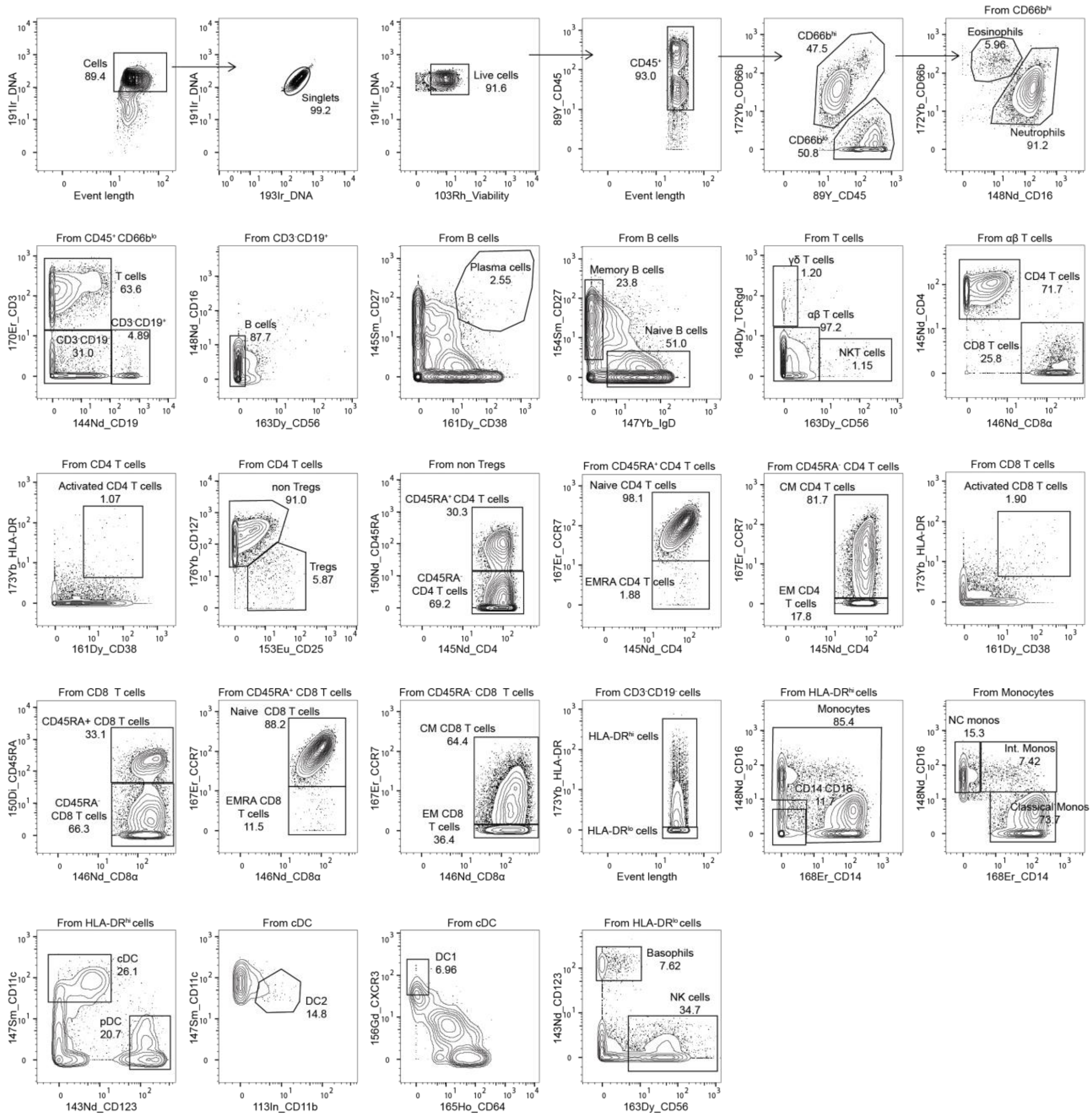


**Supplementary Figure 7. COVID-19 whole blood CyTOF gating strategy.** Manual gating verification of immune cell populations in whole blood of COVID$^-$ volunteers and COVID$^+$ patients. Percent of parent populations shown. NKT cells, natural killer T cells; Tregs, T regulatory cells; CM, central memory; EM, effector memory; EMRA, effector memory re-expressing CD45RA; NC monos, non-classical monocytes; Int. monos, Intermediate monocytes; Classical monos, Classical monocytes; cDC, conventional dendritic cells; DC1, conventional type I DC; DC2, conventional type II DC; pDC, plasmacytoid DC; NK cells, natural killer cells.

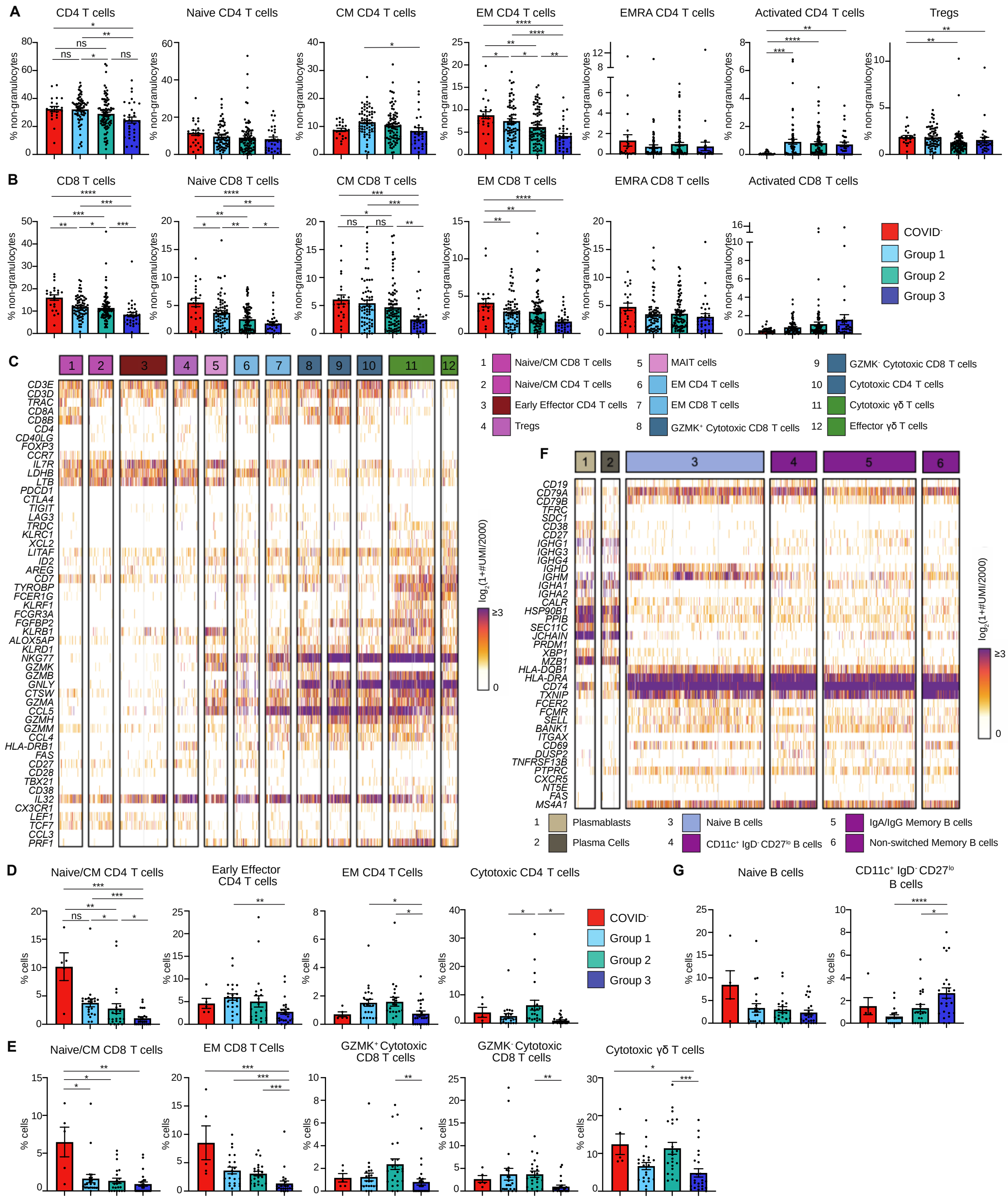

**Supplementary Figure 8. High dimensional characterization of circulating lymphocytes in COVID-19. (A and B)** The frequency of CD4 **(A)** and CD8 **(B)** T cell populations (% non-granulocytes) in whole blood by Olink group were measured by CyTOF and separated by Olink group(*n=214*). **(C)** The heatmap shows UMI counts of selected genes from T cell scRNAseq clusters from PBMC. Cell frequencies are shown as percent of cells for select CD4 **(D**), CD8 and γδ T cell **(E)** scRNAseq clusters (*n=75*). **(F)** The heatmap shows UMI counts of selected

genes from B cell scRNAseq clusters from PBMC. **(G)** scRNAseq cell frequencies are shown as percent of cells for select B cell clusters (*n=75*). For bar graphs, each dot represents a patient sample. Bars indicate the mean and error bars indicate the standard error of the mean (SEM). COVID$^{-}$ samples were obtained from healthy volunteers (A to G). Statistical significance in (A, B, D, E, and G) were determined by Kruskal-Wallis followed by multiple comparisons test with false discovery rate correction; ns, not significant; *q<0.05; **q<0.01; ***q<0.001; ****q<0.0001.

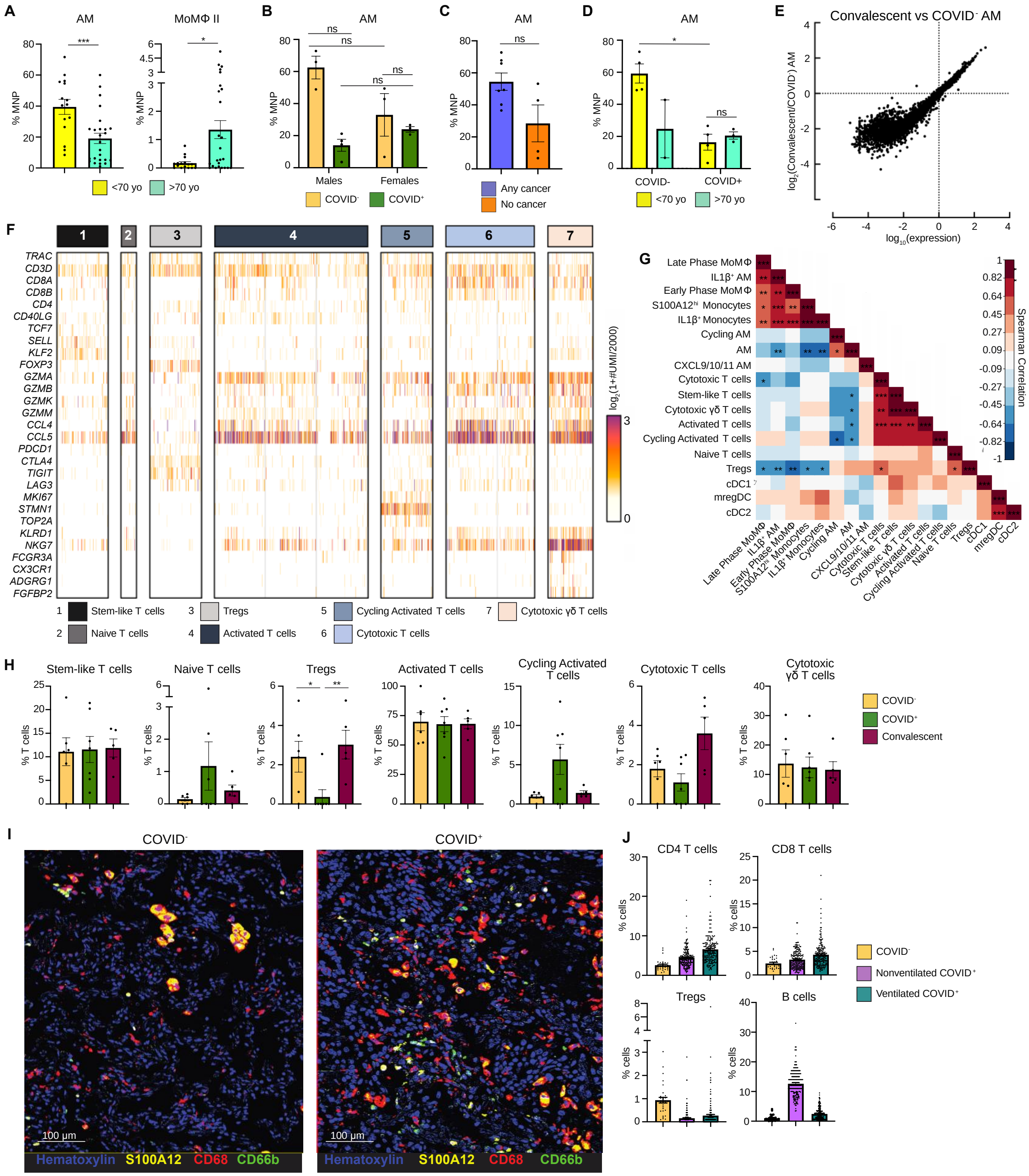

**Supplementary Figure 9. High dimensional characterization of immune cells in BAL (A)** Alveolar macrophage (AM) and Monocyte-derived macrophages-II (MoMΦ II) frequencies are shown as percent of mononuclear phagocytes (MNP) based on scRNAseq analysis of normal lung tissue from untreated early stage non-small cell lung cancer patients stratified by age ( greater than 70 or less than 70 years old)(*n=40*). **(B)** Shown is a comparison of AM in BAL as

percent of MNP by sex in COVID$^-$ and COVID$^+$ patients (*n=13*). **(C)** Comparison of AM in BAL is shown as percent of MNP in COVID$^-$ and convalescent patients stratified by cancer diagnosis (*n=11*). **(D)** Comparison of AM in BAL is shown as percent of MNP by age (greater than 70 or less than 70 years old) in COVID$^-$ and COVID$^+$ patients (*n=13*). For bar graphs in (A to D), each dot represents a patient sample. Statistical significance (A to D) was determined by Mann-Whitney test; ns, not significant, *p<0.05, ***p<0.001. **(E)** Differential gene expression between convalescent and COVID$^-$ AM is shown. **(F)** The heatmap shows UMI counts of selected genes from T cell scRNAseq clusters from BAL. **(G)** A matrix of Spearman correlation coefficients between identified scRNAseq BAL clusters is shown. *p<0.05, **p<0.01, ***p<0.001. **(H)** scRNAseq T cell cluster frequencies are shown as percent of T cells from BAL. Each dot represents a patient sample (*n=18*). Statistical significance was determined by Kruskal-Wallis test followed by multiple comparisons test with false discovery rate correction; *q<0.05; **q<0.01. **(I)** Overlaid, pseudocolored MICSSS images are shown; samples of COVID$^+$ and COVID$^-$ lungs that were stained for S100A12, CD68, CD66b, and Hematoxylin (*n=5*). Granulocytes were defined as CD66b$^+$ cells. **(J)** Quantification of lymphocytes in MICSSS images shown as percent of cells. CD4 T cells were defined as CD3$^+$ CD8$^-$ cells, CD8 T cells were defined as CD3$^+$ CD8$^+$ cells, Tregs were defined as CD3$^{+.}$ CD8$^-$ Foxp3$^+$ cells, B cells were defined as CD20$^+$ cells. Each dot represents the quantification of a single MICSSS region of interest. For all bar graphs, bars indicate the mean and error bars indicate the SEM. COVID$^-$ and convalescent samples in (B to D and H) were obtained from Mount Sinai Hospital patients.

**Supplementary Table 1. Mount Sinai Hospital Disease Severity Classification.** $SpO_2$, oxygen saturation; CXR, chest X-ray; CrCl, Creatinine Clearance; ALT, Alanine aminotransferase; ULN, upper limit of normal; RRT, renal replacement therapy; WHO, World Health Organization

| **Disease Severity** | | |
|---|---|---|
| Moderate COVID-19 | $SpO_2$<94% on RA or pneumonia by CXR, ≦6 L/min O2 support | WHO clinical progression. score: 4-5 |
| Severe COVID-19 | >6L/min $O_2$ support, (-) pressors, CrCl>30 mL/min, ALT<5x ULN | WHO clinical progression score: 6-7 |
| Severe COVID-19 with EOD | >6 L/min $O_2$ support, (+) pressors, CrCl<30 mL/min or new RRT or ALT>5x ULN | WHO clinical progression score 8-9 |

**Supplementary Table 2. Clinical information for PBMC scRNAseq samples.**

| Participant ID | Timepoint | Age | Sex | Days PSO | Disease severity | Olink Group | Olink Cluster | Final Clinical Outcome |
|---|---|---|---|---|---|---|---|---|
| Subj_21b7ab7c | T1 | 28 | M | n/a | COVID$^-$ | n/a | n/a | Survived |
| Subj_5aabb512 | T1 | 25 | F | n/a | COVID$^-$ | n/a | n/a | Survived |
| Subj_a62483bb | T1 | 58 | M | n/a | COVID$^-$ | 1 | 12 | Survived |
| Subj_511a0966 | T1 | 56 | F | n/a | COVID$^-$ | 1 | 12 | Survived |
| Subj_8df7db09 | T1 | 50 | F | n/a | COVID$^-$ | n/a | n/a | Survived |
| Subj_8aeb8455 | T1 | 51 | M | n/a | COVID$^-$ | n/a | n/a | Survived |
| Subj_85b4a5d2 | T1 | 68 | | n/a | n/a | 1 | 15 | Deceased |
| Subj_449c6103 | T1 | 65 | M | 7 | EOD | 2 | 9 | Survived |
| | T12 | | | 19 | Moderate | 1 | 13 | |
| Subj_f5fc1a21 | T1 | 65 | F | 3 | Moderate | 1 | 13 | Survived |
| | T4 | | | 8 | Moderate | 1 | 14 | |
| Subj_d650fa11 | T1 | 80 | M | 32 | EOD | 2 | 6 | Survived |
| | T4 | | | 35 | Moderate | 1 | 13 | |
| | T8 | | | 39 | Moderate | 1 | 15 | |
| Subj_6968e8c2 | T1 | 83 | F | 16 | Severe | 1 | 13 | Survived |
| | T4 | | | 19 | Moderate | 2 | 6 | |
| Subj_175135fe | T12 | 89 | M | 19 | Severe | 1 | 13 | Survived |
| Subj_cb70545d | T1 | 39 | F | 28 | Moderate | 1 | 14 | Survived |
| Subj_6ac7cb7b | T1 | 59 | M | 12 | Moderate | 1 | 14 | Survived |
| | T4 | | | 15 | Moderate | 2 | 7 | |
| | T8 | | | 21 | Moderate | 1 | 14 | |
| Subj_f3900bdf | T13 | 68 | M | 37 | Moderate | 1 | 14 | Survived |
| Subj_fc557f8f | T4 | 39 | M | 16 | Severe | 1 | 15 | Survived |
| | T8 | | | 20 | Severe | 1 | 15 | |
| Subj_b5095d08 | T4 | 47 | F | 11 | Severe | 1 | 15 | Survived |
| | T8 | | | 16 | Severe | 1 | 15 | |
| Subj_2b440626 | T1 | 53 | M | 5 | Moderate | 3 | 1 | Survived |
| | T4 | | M | 8 | Moderate | 1 | 15 | |
| Subj_7cf00ea9 | T1 | 66 | M | 5 | Moderate | 2 | 8 | Survived |
| | T4 | | | 8 | Severe | 2 | 8 | |
| | T12 | | | 17 | Moderate | 2 | 8 | |
| | T13 | | | 27 | Moderate | 1 | 15 | |
| Subj_a56fd839 | T4 | 69 | F | 20 | Moderate | 1 | 15 | Survived |
| Subj_de46f293 | T12 | 71 | M | 17 | Severe | 1 | 15 | Survived |
| Subj_b18b35ec | T1 | 70 | M | 11 | Moderate | 2 | 8 | Survived |
| | T4 | | | 17 | n/a | n/a | n/a | |
| Subj_4f2957ee | T1 | 78 | M | 29 | Moderate | 2 | 6 | Deceased |
| Subj_e34fa597 | T1 | 89 | F | 18 | Moderate | 3 | 5 | Survived |
| | T4 | | | 21 | Moderate | 2 | 6 | |
| Subj_318dda84 | T1 | 20 | M | 5 | EOD | 3 | 3 | Survived |
| | T4 | | | 9 | Moderate | 2 | 7 | |
| | T8 | | | 12 | Moderate | 2 | 7 | |
| Subj_224dc393 | T1 | 70 | M | n/a | Moderate | 2 | 7 | Survived |

| | | | | | | | | |
|---|---|---|---|---|---|---|---|---|
| | T4 | | | n/a | Moderate | 2 | 7 | |
| Subj_7213a9b9 | T1 | 69 | M | 16 | Severe | 2 | 8 | Survived |
| | T8 | | | 23 | EOD | 3 | 5 | |
| Subj_8557d073 | T1 | 32 | M->F | 7 | Severe | 2 | 9 | Survived |
| | T4 | | | 13 | Severe | 3 | 1 | |
| Subj_464a28c7 | T1 | 69 | F | 7 | Moderate | 2 | 9 | Survived |
| | T4 | | | 10 | EOD | 3 | 4 | |
| | T12 | | | 20 | EOD | 3 | 4 | |
| | T13 | | | 29 | EOD | 2 | 9 | |
| Subj_245ec65e | T8 | 80 | M | 21 | Moderate | 2 | 9 | Survived |
| Subj_74d9ca69 | T1 | 47 | M | 23 | EOD | CKD | 10 | Deceased |
| | T4 | | | 26 | EOD | CKD | 11 | |
| | T8 | | | 30 | EOD | 3 | 2 | |
| | T12 | | | 37 | EOD | 3 | 2 | |
| Subj_972d9455 | T8 | 74 | F | n/a | EOD | 3 | 5 | Deceased |
| Subj_05fd9717 | T1 | 94 | F | 10 | EOD | 3 | 2 | Deceased |
| Subj_52c9ea6b | T1 | 68 | F | 14 | Severe | 3 | 3 | Deceased |
| Subj_626935c9 | T4 | 84 | F | n/a | EOD | 3 | 5 | Deceased |
| Subj_d683ade0 | T4 | 66 | F | 9 | EOD | 3 | 2 | Survived |
| | T8 | | | 12 | Moderate | 3 | 2 | |
| | T12 | | | 16 | Moderate | 3 | 4 | |
| Subj_3fb3ab81 | T1 | 70 | F | 3 | EOD | 3 | 4 | Survived |
| | T4 | | | 6 | Moderate | 3 | 2 | |
| Subj_e3d686b8 | T1 | 65 | M | 24 | EOD | 3 | 3 | Survived |
| | T4 | | | 27 | EOD | 3 | 3 | |
| Subj_17abecb0 | T4 | 69 | F | 6 | EOD | 3 | 4 | Survived |
| | T8 | | | 11 | EOD | 3 | 4 | |
| | T12 | | | 21 | Severe | 3 | 3 | |
| | T13 | | | 25 | Severe | CKD | 11 | |
| Subj_ea04d4fd | T4 | 59 | F | 16 | Moderate | CKD | 10 | Deceased |
| | T8 | | | 20 | Moderate | CKD | 10 | |
| | T12 | | | 23 | Moderate | CKD | 10 | |
| Subj_1b1361b3 | T1 | 33 | F | 9 | Moderate | CKD | 10 | Survived |
| Subj_79c38c5e | T4 | 57 | F | 36 | Moderate | CKD | 11 | Survived |
| | T8 | | | 40 | Moderate | CKD | 11 | |

**Supplementary Table 3. Clinical information for BAL samples.** Conv, convalescent; PI, post intubation; BAL Dx, BAL diagnosis

| Participant ID | COVID Status | Outcome | Days PSO | Days PI | Age | Sex | Smoking Status | BAL Dx |
|---|---|---|---|---|---|---|---|---|
| Subj_4a4e5a82 | + | Survived | 17 | 2 | 56 | M | Former | n/a |
| Subj_dc557cb4 | + | Survived | 41 | 2 | 51 | M | Never | n/a |
| Subj_b7bdb86b | + | Deceased | 22 | 1 | 72 | M | Never | n/a |
| Subj_8e88aa3b | + | Deceased | 18 | 0 | 94 | F | Never | n/a |
| Subj_0f26ec77 | + | Survived | 21 | 3 | 51 | F | n/a | n/a |
| Subj_67573a91 | - | Survived | | | 61 | F | Former | Neuroendocrine carcinoma metastasis |
| Subj_33ab3911 | Conv | Survived | | | 44 | M | Former | Metastatic lung adenocarcinoma |
| Subj_1391ab64 | Conv | Survived | | | 51 | M | Former | Carcinoid tumor |
| Subj_5daa15c9 | - | Survived | | | 70 | F | Former | Mild chronic inflammation; squamous metaplasia |
| Subj_38c320d9 | - | Survived | | | 65 | M | Former | Lung squamous cell carcinoma |
| Subj_00f88702 | - | Survived | | | 61 | M | Former | Lung squamous cell carcinoma |
| Subj_1eaf71d6 | Conv | Survived | | | 72 | M | Current | Benign lung nodules |
| Subj_7b5a9762 | - | Survived | | | 59 | M | Current | Palatine tonsil squamous cell carcinoma metastasis |
| Subj_4766af57 | Conv | Survived | | | 61 | M | Former | Renal cell carcinoma metastasis |
| Subj_2a21c17d | Conv | Survived | | | 62 | F | Never | Inflammatory Lung Nodules |
| Subj_5bf84fe1 | - | Survived | | | 71 | F | Former | Interstitial Pulmonary Fibrosis |

**Supplementary Table 4. Clinical Information for lung autopsy samples.** PMI, post mortem interval; AHRF, acute hypoxic respiratory failure; 2/2, secondary to

| Participant ID | Age | Sex | PMI (hours) | Days PSO | Days hospitalized | Intubated | Cause of Death |
|---|---|---|---|---|---|---|---|
| MA-20-120 | 59 | F | 5.5 | 13 | 8 | No | AHRF 2/2 COVID-19 |
| MA-20-149 | 77 | F | 7.5 | 25 | 22 | Yes | AHRF 2/2 COVID-19 |
| MA-20-123 | 61 | M | 3 | 29 | 48 | Yes | AHRF 2/2 COVID-19 |
| MA-20-81 | 57 | F | N/A | 4 | 4 | No | AHRF 2/2 COVID-19 |

**Data File S1. Mount Sinai COVID-19 Biobank Cohort Clinical Characteristics by clinical severity.** Statistical significance for all categorical variables was determined by Chi square test followed by two-sided Fisher's exact test between severity groups. Statistical significance for quantitative variables was determined by Kruskal-Wallis test with multiple hypothesis correction by Dunn's multiple comparisons test.

**Data File S2. Mount Sinai COVID-19 Biobank Cohort Clinical Characteristics by Olink Group.** Statistical significance for all categorical variables was determined by Chi square test followed by two-sided Fisher's exact test between Olink groups. Statistical significance for quantitative variables was determined by Kruskal-Wallis test with multiple hypothesis correction by Dunn's multiple comparisons test.

**Data File S3. Treatment effects on Olink cytokine concentrations.** Multiple Welch's t-tests with false discovery rate (FDR) adjustment were used to compare steroid (dexamethasone, methylprednisolone, prednisone, prednisolone) treatment for Group 2 at T1 (Sheet 1), or at last patient timepoints (Sheet 2), or for Group 3 at T1 (Sheet 3), or at last patient timepoints (Sheet 4). Multiple Welch's t-tests with FDR adjustment were used to compare Norepinephrine treatment for Group 3 at T1 (Sheet 5), or at last patient timepoints (Sheet 6). Multiple Welch's t-tests with FDR adjustment were used to compare Heparin treatment for Group 2 at T1 (Sheet 7), or at last patient timepoints (Sheet 8), or for Group 3 at T1 (Sheet 9), or at last patient timepoints (Sheet 10). Multiple Welch's t-tests with FDR adjustment were used to compare Enoxaparin treatment for Group 2 at T1 (Sheet 11), or at last patient timepoints (Sheet 12), or for Group 3 at T1 (Sheet 13), or at last patient timepoints (Sheet 14).

**Data File S4. Whole blood CyTOF cell counts and frequencies.**

**Data File S5. PBMC scRNAseq cell counts.**

**Data File S6. BAL scRNAseq cell counts.**
